# Assessing and measuring financial sustainability model of the Spanish HIV HGM BioBank

**DOI:** 10.1186/s12967-019-02187-w

**Published:** 2020-01-06

**Authors:** Irene Consuegra Fernández, Isabel García Merino, María Ángeles Muñoz-Fernández

**Affiliations:** 1 Immunology Section, Molecular ImmunoBiology Laboratory, Hospital General Universitario Gregorio Marañón and Instituto Investigación Sanitaria Gregorio Marañón, C/Doctor Esquerdo 46, 28007 Madrid, Spain; 2 Spanish HIV HGM BioBank, Madrid, Spain; 3 Networking Research Center on Bioengineering, Biomaterials and Nanomedicine (CIBER-BBN), Instituto de Salud Carlos III, 28029 Madrid, Spain

**Keywords:** BioBanks, Personalized medicine, Future pathologies, Financial BioBank sustainability, Cost recovery model

## Abstract

**Background:**

The Spanish HIV HGM BioBank is of great relevance for basic and clinical investigation, and for those groups trying to establish large networks focused on investigation on specific clinical problems. The collection of different types of samples from HIV-infected individuals is the beginning of the chain of translational investigation, starting in 2004 a prospective national HIV BioBank that expanded in 2009 a local node (HGM: Hospital Gregorio Marañón) for diverse pathologies and clinical networks, not only in adults but also in paediatric patients, becoming the Spanish HIV HGM BioBank. Our main objective is to find a general criteria and analytical tools to widespread its economic management to assure their sustainability and the future exploitation of the extreme high valuable biomaterial they custody.

**Methods:**

The Spanish HIV HGM BioBank was created with the aim of contributing to advance understanding of different pathologies through the transfer, management, register, processing, cryopreservation and cession of biological material from patients, always for research purposes and under conditions that guarantee its usefulness in current studies and future research that may appear as knowledge evolves. In this study, we have developed a policy for financial control and recovery costs of the Spanish HIV HGM BioBank.

**Results:**

Actually, Spanish HIV HGM BioBank guards 413,747 vials of 46,594 samples from 16,210 donors with various prospective longitudinal study type of samples. Interestingly, more than 7907 of these samples are now used in 28 national and international investigation projects and clinical trials. One of the objectives of this study is to develop an economic plan that you get future projects, design of acceptance or rejection keys, have internal investment limits, minimum recovery needs in short/medium term, deviation detection system and a register of capital recovery by period and type of service for the Spanish HIV HGM BioBank.

**Conclusion:**

Our model can help BioBanks that do not have a costs recovery model to design it, as well as to detect improves and functional revisions to those experienced in this field.

## Background

The development of personalized medicine is becoming a reality, creating the very best treatments thanks to research in genetic, genomic and phenotypic factors. Transferring personalized medicine to clinical practice requires research with large and varied collections of biological samples and associated clinical data. The BioBanks are research-supportive platforms that act as a link among the donors, clinics and researchers [1].

BioBanks are complex to run from an operational and administrative point of view and must adjust to and control the intrinsic and exclusive features associated with their work, highlighting: (1) uncertainty in the use of bio-resources, maintenance of spaces for the storage, a proper control of the stocks of biomaterial, maintenance’s costs and measures to avoid accumulating material; (2) funding for BioBanks, which are mostly coming from the public administration, and difficulty in financial outlining, foreseeing, growth or control of BioBanks.

HIV BioBank was created in 2004 as a national platform with the aim of contributing to advance in understanding of HIV infection. Its development increased in 2009 with the set-up of a local node of different pathologies, mostly paediatrics, always for research purposes and under conditions that guarantee its usefulness in current studies and future research that may appear as knowledge evolves (HGM node). Actually, HIV HGM BioBank receives samples from 14 HIV cohorts/studies/clinical trials and 15 collections of different pathologies in the HGM local node. Spanish HIV HGM BioBank guards 413,747 vials of 46,594 samples from 16,210 donors with various prospective longitudinal study type of samples. Since its creation more than 58,056 aliquots from 16,588 samples have been transferred in 139 national and international investigation projects and clinical trials. This activity allowed the publication of 179 articles derived of the use of its bioresources (Additional file 1).

A significant fact is the non-specific training in economic matters of the managers, the majority of whom, are researchers with university degrees in science fields. The absence of commonality in the economic management of the BioBanks and the difficulty in achieving sustainability and control, worries their professionals because it is challenging to design a system that can apply to all [2–5]. Computer resources to control costs do not exist, to the limit of our knowledge. There are very few software tools available for the economic control of BioBanks, and they are not open access (BEMT) or requires subscription (BRC), and neither of them are useful for any BioBank. Therefore, what we need is a design of personalized budget, control of sample stocks and short, medium and long-term financial planning to compensate for the necessity of staff training in economic management [6–9].

BioBanks manage their economic resources depending on whether their administration is private, public, national, regional, etc. [10]. The only horizontal directive is the requirement to be exempt from profit. Taking that into account, each BioBank can set up its own policy for recovering costs, as part of its strategic plan [11]. Therefore, we detail in this article the measures and tools developed by the Spanish HIV HGM BioBank to determine the funding needed for its operation, forecasts, elements of control and escalation of prices [12, 13]. Our goal is to achieve BioBank’s economic self-sustainability and ensure its function in the medium and long-term, while maintaining top standards of excellence in samples quality and clinical data associated.

## Materials and methods

The first step is to established of a cost analysis model. To introduce an analysis tool that settles the expenses incurred by the BioBanks [14–17]. This analysis studies retrospective data, draws up service budgets, and forecasts prospective collections according to demand [18, 19]. We considered different economic models for this goal: (1) partial costs system: includes only direct costs involved in the production process based on developed activities. Indirect production costs are charged directly to the income statement. This system was rejected because it does not include fixed and large budgetary items. (2) Complete cost system: attributes all direct and indirect costs to final price, without differentiating between fixed and variables. This method is not valid for BioBanks because the costs of later storage are excluded. (3) Standard cost system: is based on the final costs of a product to set objectives in terms of price competitiveness. This is not appropriate as one of our objectives is to maximize excellence in quality, not in prices. (4) ABC-activity based cost system: enables indirect costs to be assigned and distributed in accordance with the activities carried out as they generate the costs. A cost involves a chain of activities, productive processes, which may be included in all the actions that the BioBank needs to carry out until an activity is completed, minimizing the factors that do not increase expenses. To study and implement ABC estimation we use “Model of Costs Analysis for BioBanks” drawn up by the working group on costs analysis of the Spanish National Network of BioBanks (website http://www.redbiobancos.es/).

### Determining the parts of the productive process of the Spanish HIV HGM BioBank

(a) Analyzing the productive processes to identify the stages: (A) cost per unit of stored product; (B) cost per sample: storage and estimation of retrospective samples; (C) cost for reception and dispatch; (D) cost per order: activities carried out after donating the samples; (E) technical services: cellular, molecular and histological techniques included in the catalogue; and (F) services free of biomaterial: teaching, legal, ethics, consultancy… (b) Establishing costs targets: each stage of the productive process A, B, C, D are studied on the basis of cost/aliquot according to the type of sample. Processes E and F are based on the cost/service and/or the technique used. (c) Identifying resources that are consumed in each stage. Analysis of: reagents, perishables, courier, equipment, personnel, and other costs (electricity, water, cleaning, telephone). (d) Assigning costs to the stages of the process. Selection of costs that are involved in each part of the process. The expenses in each stage in the Spanish HIV HGM BioBank are due to A: costs of personnel, perishables, reagents, equipment, courier and other costs. B: costs of personnel, perishables, equipment and other costs. D: costs of personnel, perishables and other costs. E: costs of personnel, reagents, perishables, equipment and other costs and F: costs of personnel, perishables and other costs. (e) Implementation and calculation of main costs: Customized budgets are prepared and registered according to our unit costs. These calculations show the price/aliquot of retrospective samples (A + B + C + D) or the investment needed to start a new collection of indeterminate (A + B + C + F) or pre-stablished (A + B + C + D + F) duration.

### Criteria for distribution of indirect costs

In the Spanish HIV HGM BioBank indirect cost are personnel, equipment and other costs. In the case of personnel, the cost of the hours/product/service is assigned to each one of the productive stages. In equipment the applicable criterion is the usage time for each device, each stage of the process and redemption. Finally, the costs of electricity, water, cleaning services and telephone, is assigned to each product (cost/3 years average production), and added as “other costs” [6, 7].

### Determining the value of the stocks

The cryopreserved material does not have donation, expiry or safety-of-use dates. The samples do not increase in value over time, but they gradually accumulate costs. Furthermore, the volume of sample transfers is very unstable, due to the fluctuating number of requests and the possibility to transfer the same sample to a single project or many different ones. For all these reasons, it is not possible to make a reliable estimation, with small variations, of the BioBank’s performance over the following year. To determine the value of the stock we carried out a study based on (1) cost/sample for the production process A and cost/sample for B in 1 year; (2) determining the number of aliquots stored in each year since 2004 until June 2018 (discounting donated samples). The global costs of 1 × 2, accounting for the time stored, enables us to know the overall value of the stocks [18, 19].

### Costs recovery policy

The Spanish HIV HGM BioBank has designed a cost recovery policy (CRP) based on different scales of discount depending on the type of service requested: Members of our hospital (HGM) and Spanish AIDS Research Network (RED RIS) − 70%, public funds − 50%, clinical trials − 35%, firms 0%.

### Analysis of sustainability and financial projections

To analyze the current development of the RCP and the design of the Spanish HIV HGM BioBank’s short- and medium-term financial projections, we make a register of all the applications received that have been responded with a personalized budget. This analysis has been mandatory for all Spanish HIV HGM BioBank customers since 2016. The register includes in chronological order all the applications divided into the following categories: Collection: setting up of a cohort of prospective samples in accordance with the regulatory access. Clinical study: setting up of a collection of prospective samples for a study subsidized through a public body. Project: assignment of retrospective samples to a project. Clinical trial: assignment of retrospective/prospective samples for clinical trials. Course: teaching services. Other services: activities included in the Spanish HIV HGM BioBank catalogue.

For each request processed, we recorded data on gross, incurred gross, budgeted and granted costs. Gross costs (GC): total sum of all the possible costs needed to develop a request without discounts and taking into consideration all the direct and indirect costs of the budgetary items involved. This amount reflects the complete investment that the Spanish HIV HGM BioBank needs to face for each serviced requested. It is a theoretical cost for prospective samples collections. For requests that involve the use of retrospective samples, part of the gross costs have already been paid by the Spanish HIV HGM BioBank. Only when the budget is approved by customers, costs are back paid. Incurred gross costs (IGC): these are costs resulting from the implementation of accepted budgets, without discounts and taking into consideration all the direct and indirect costs of all the budgetary items. This amount reflects the real investment that each of the requests means for the Spanish HIV HGM BioBank. Budgeted costs (BC): depending on the applicant and the funding body, the Spanish HIV HGM BioBank applies a discount policy. This budget corresponds to the amount that the Spanish HIV HGM BioBank asks for the work carried out. The budgeted costs are also theoretical until the client accepts it, and signs the MTA. Granted costs (GDC): the amount that the client/researcher finally signs for the services offered. It may not be the same as the forecasted in the budget due to negotiations with the customer or specific agreements with the Spanish HIV HGM BioBank management.

The annual grouping of each block gives an idea of the real whole amounts managed by the Spanish HIV HGM BioBank, considering theoretical costs that could have been incurred, real income received, and amounts paid by the Spanish HIV HGM BioBank. The study of this structured data gives a descriptive image of the development of the Spanish HIV HGM BioBank’s funds and tries to provide a road map, find diversions and corrects them ahead.

Summing up, we can group the costs in blocks and make a temporary or typological data analysis considering the ratios defined below. Average budget/request: accumulation of all the BC in a year divided by the requests analyzed. It gives an idea of the recovery average per year. Gross/budgeted costs ratio: summary of all the GC in a year divided by all the BC. It shows us the gap between the costs that may have been invested by the Spanish HIV HGM BioBank and the income expected from said activity. This ratio is useful in order to know if the discount policy designed by management fits in with the real financial needs. In this way, the discount scale for the next cycles is adjusted on the basis of information gained from previous years. Recovery from budgets (GC/GDC): This ratio comes from the sum of the theoretical GC and the real income obtained in a full financial year. It gives an idea of the success achieved in the negotiation with the client/researcher. If the collaborations are not finalized, or the gross costs are very discordant with the final budget agreement, the costs of carrying out the request and/or the negotiation process require supervision by the management and improvement. Recovery from investment (IGC/GDC) this ratio provides information on the returns from the investment. This ratio is influenced by the typology of the requests received.

**Fig. 1:**
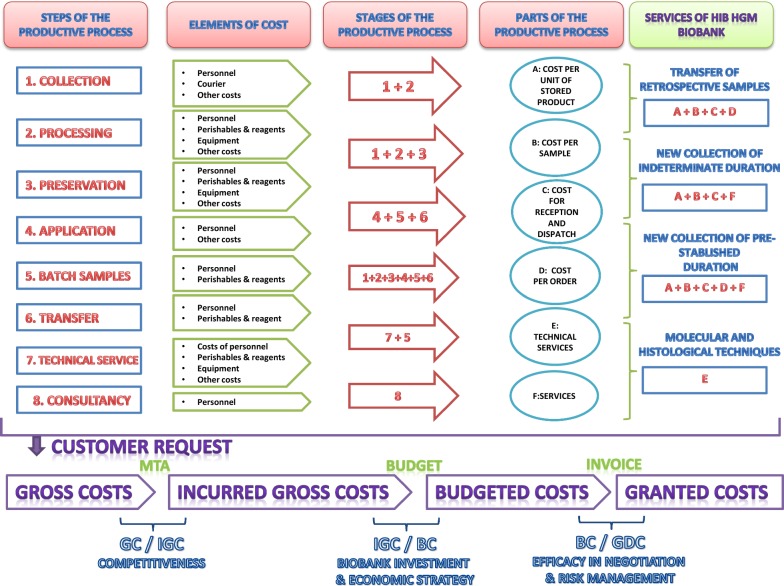
Flow chart for different sections of the financial methodology of Spanish HIV HGM BioBank, including its productive process, services available for customers and analysis of the costs and investments required for each step

The annual measuring provides chronological information of the economic development from 2013 to the present, as well as global averages for each ratio. Averages and ratios give an objective measurement of the activity and efficiency in the economic management of the Spanish HIV HGM BioBank’s resources and, based on it, we can make predictions and design contingency plans for short and medium term (Fig. 1).

## Results

### Implementation of an ABC model

Since 2012, the Spanish HIV HGM BioBank carries out an exhaustive economic-financial analysis of its activity as required by the NRB according to Royal Decree 1716/2011, of 18th November legal dispositions. Any costs related to its activity, whether direct or indirect, are assigned to the productive process stages A, B, C, D, E and F, previously defined. This study generates a catalogue of prices that can be consulted on the website: http://hivhgmbiobank.com/researchers-area/our-services/techniques/?lang=en.

The value of the Spanish HIV HGM BioBank’s stock is also contemplated in this ABC analysis. To obtain this information we calculate both the storage time of each aliquot and the costs derived from its storage in one year (electricity, CO_2_ and cooling equipment), so we can assign to each aliquot the costs incurred from the moment of its processing up to the present day. The value of the Spanish HIV HGM BioBank’s stock in the moment of its authorization by ISCIII amounted to 62.267.822 €.

### Implementation of costs recovery policy

**Fig. 2:**
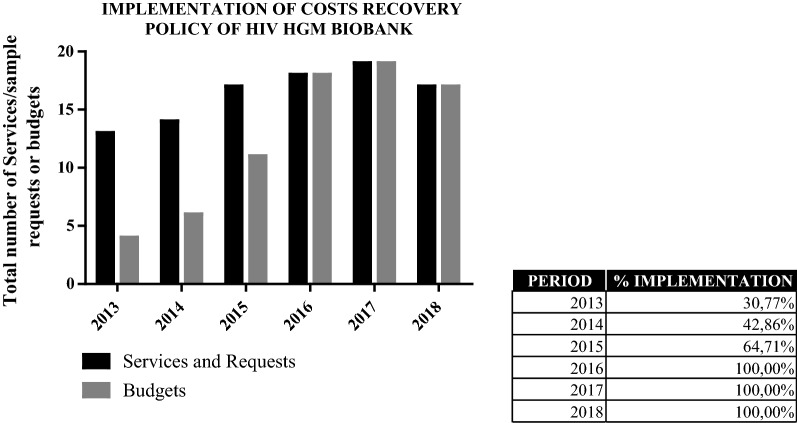
Implementation of CRP and efficacy of its dissemination among the Spanish HIV HGM BioBank’s customers (2013–2018)

The implementation of sustainability policy has been carried out in a progressive way since 2013. The Spanish HIV HGM BioBank designed and developed an information campaign for its users before the CRP was established, which has been maintained since then. This strategy will cover the new economic requirements without prejudice for the scientific community and minimising its impact. Clients are informed about the tariffs assigned to the biomaterial and the legal directives associated. They are urged to include the BioBank’s budgetary amount when they apply to financial bodies. This system of spreading the CRP led, in the first years, to only responding with a budget to those applications received from customer with adequate funding. Gradually, the CRP has been included in the Spanish HIV HGM BioBank’s usual procedures of assessment, in parallel with the criteria of the scientific and ethical committees (Fig. 2). Explained in an easy and sequential outline this process of economic analysis has been described in the document “Flow chart for HIV HGM BioBank’s economic proposals” available in its website, http://hivhgmbiobank.com/researchers-area/?lang=en. Moreover, the procedure of analysis and forecast of the CRP has been presented in national and international meetings and conferences.

### Evolution of costs recovery policy

Since 2013 all the projects analyzed financially are registered. The information included from each request is GC, IGC, BC and GDC.

**Fig. 3:**
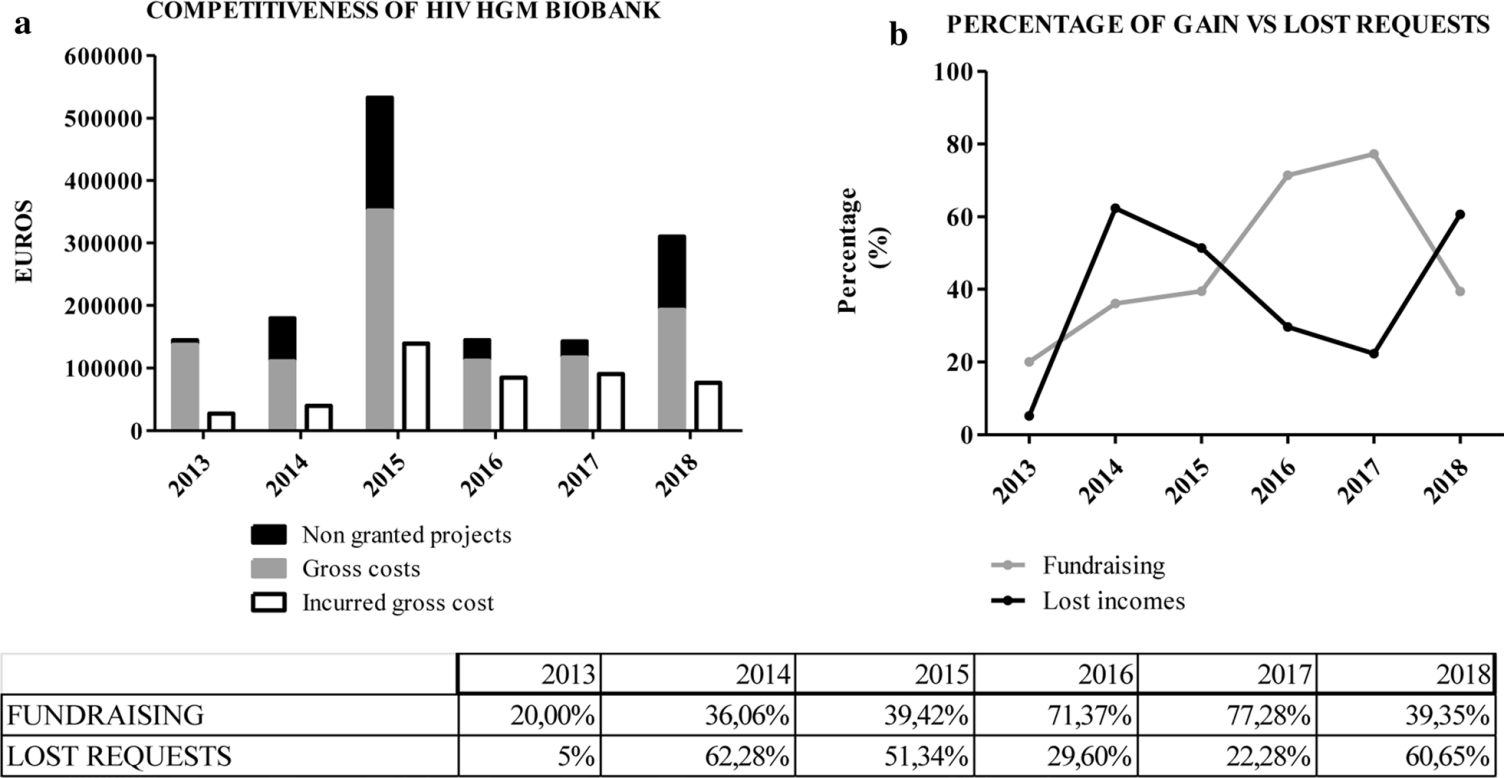
**a** Competitiveness of HIV HGM BioBank. **b** Percentage of gain vs lost request. Competitiveness evolution of Spanish HIV HGM BioBank, 2013–2018. Level of success in signing the budget. Lost requests are considered as developed in other BioBank, which indicates the success or failure in communicate Spanish HIV HGM BioBank’s strength

GC–IGC: shows the degree of competitiveness. It is interpreted as the final clients’ decision to select our Spanish HIV HGM BioBank instead of others. Research projects that request a budget before applying to funding bodies are also registered, referred as non-granted projects (Fig. 3a, b).

**Fig. 4:**
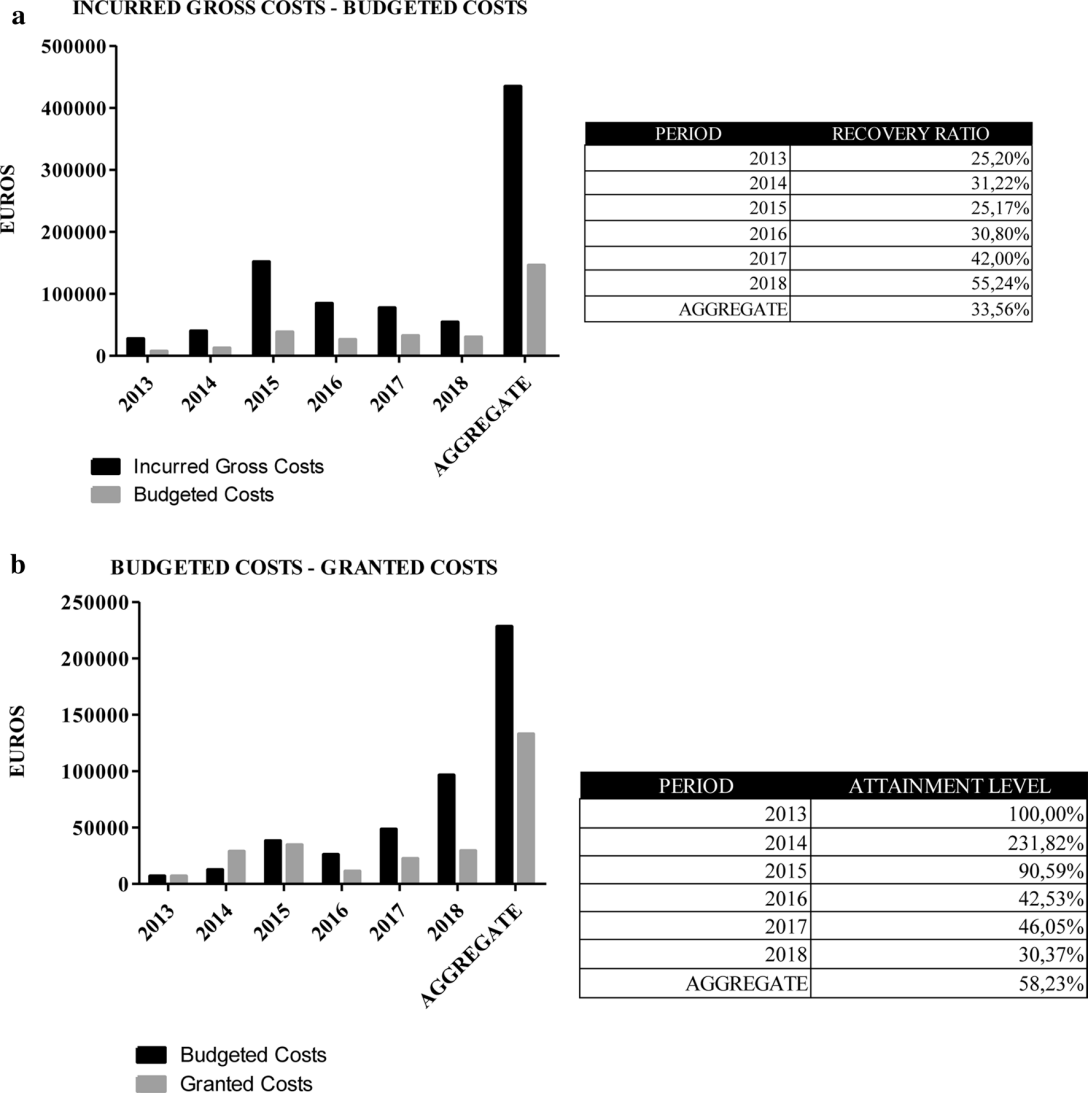
Incurred gross cost–budgeted cost.** a**: Comparative analysis between investments of Spanish HIV HGM BioBank’s capital versus volume of recovery budgeted. Budgeted cost–granted cost. **b** Negotiation success in signing the Spanish HIV HGM BioBank’s budgets. Deviations in this stage entail a need for increase the investment of own capital thus economic viability of the project and Spanish HIV HGM BioBank can be compromised

IGC–BC: informs us about the real investment needed in a given period of time to undertake a service performed (Fig. 4a). It is very important to know the economic demands and their fluctuations in previous years in order to make an estimation of the needs for future cycles. It is very complex to make financial projections in entities with the systematics and peculiarities that BioBanks exhibit. Volumes of samples received, retrospective samples donated, requests of set up prospective biological material and services are subjected neither to predictable demands nor to stable development patterns. Based on the origin of the funding of each project, Spanish HIV HGM BioBank applies a scale discount that makes the final budget more or less similar to IGC. A large gap between investment and budgets indicates that the majority of applicants are funded by public administrations. On the contrary, when this ratio is low, BioBank works mainly with private funded projects or clinical trials. These analysis helps the BioBank to decide its economic strategy, determining which niche of clients to address in order to meet its objectives in the future.

BC–GDC: provides information on the efficacy of the BioBank’s negotiation and on budgets closing (Fig. 4b). This data shows the gap between the financial needs and the real amount recovered. A large discrepancy between budgeted items poses a real risk to the financial survival of the BioBank. Therefore, this discrepancy must be an alarm for taking economic decisions in the short-term. Setting minimum limits for income and deviations in this measure is a crucial decision that management should take when starting a financial plan.

### Decision tools of the costs recovery policy

#### Estimation of operational needs

**Fig. 5:**
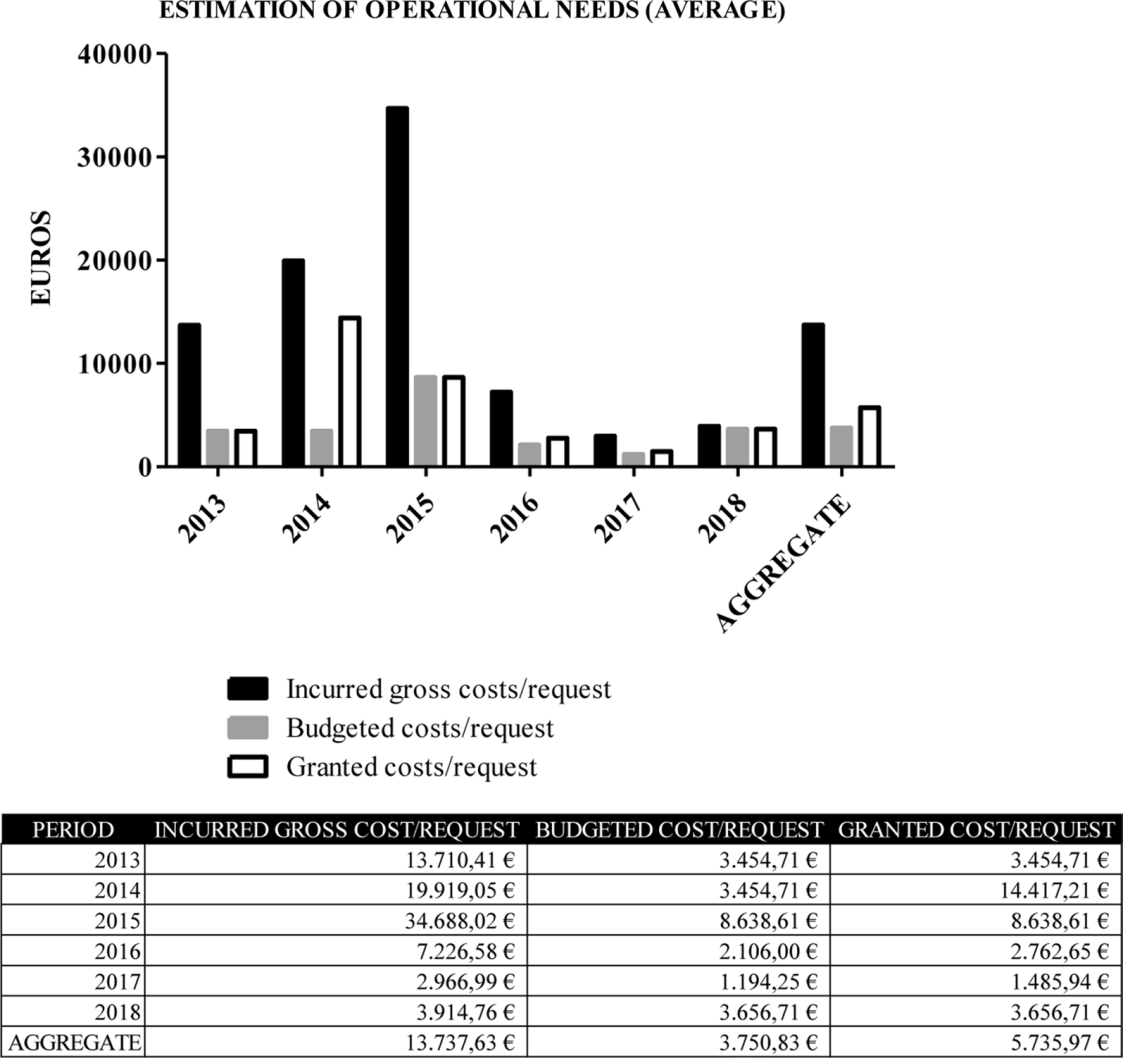
Estimation of operational needs. Averages per request by year, 2013–2018. Provides a preliminary idea of the type of services developed in a period (public or private funding)

We analyzed the measures for services performed with IGC, budgets executed and granted costs, giving a sequential picture of what happened each cycle (Fig. 5).

Although BioBanks have limited capacity for financial prediction, an analysis of what happened in previous years provides information on investment recovery parameters and future provision. The implementation of a risks management policy that rejects projects that exceed statistical alarm limits based on Levey–Jennings graphs is the best example, stemming from the central limit theorem. The CRP is under control if the results follow a normal distribution since the recorded data will form a Gaussian distribution and approximately 99.7% of the results will be will be within the limits of + 3 s [10, 11].

**Fig. 6:**
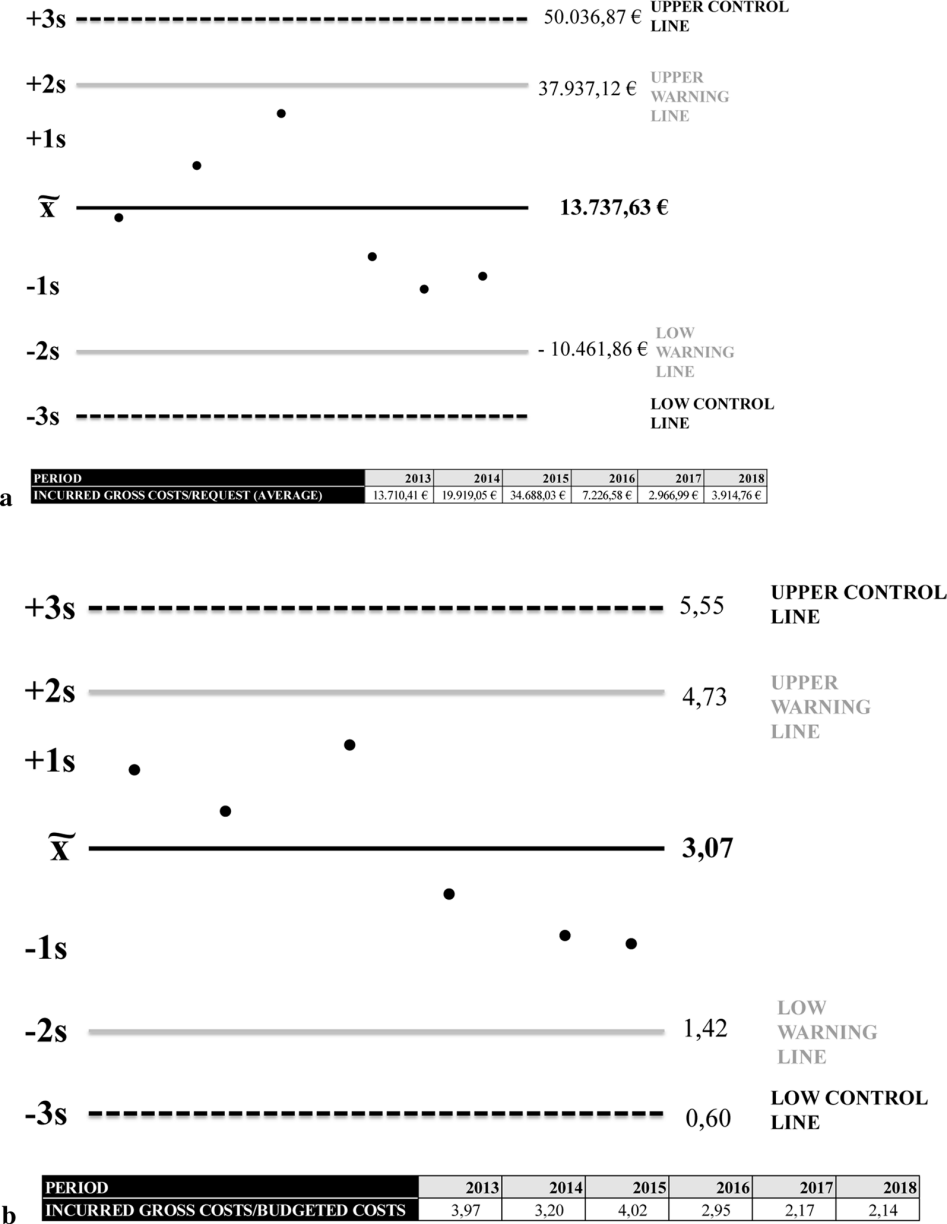
**a** Risks management policy supported in alarm limits based on Levey–Jennings graphs. Spanish HIV HGM BioBank records, and warning and control points for investments, 2013–2018. **b** Risks management policy supported in monitoring acceptable and optimal quantities of investment based on Levey–Jennings graphs. Spanish HIV HGM BioBank records with warning and control points for recovery, 2013–2018

The financial activity enables the results to be interpreted as forms of advice and control, but not necessarily as forms of rejection. Using the average and standard deviation on the average of incurred gross costs, the Spanish HIV HGM BioBank can set decision limits. However, final approval must be directed by the scientific management taking into accounts essential factors such as the relevance of a particular study, not basing the decision exclusively on economic factors. The services that exceed level + 2 s are review and studied, but request over + 3 s of the Spanish HIV HGM BioBank’s investment measure must be meticulously analyzed by the management before being accepted or rejected as a preventive check point to control economic sustainability (Fig. 6a).

#### Ratios of estimated recovery and real recovery of the investment

**Fig. 7:**
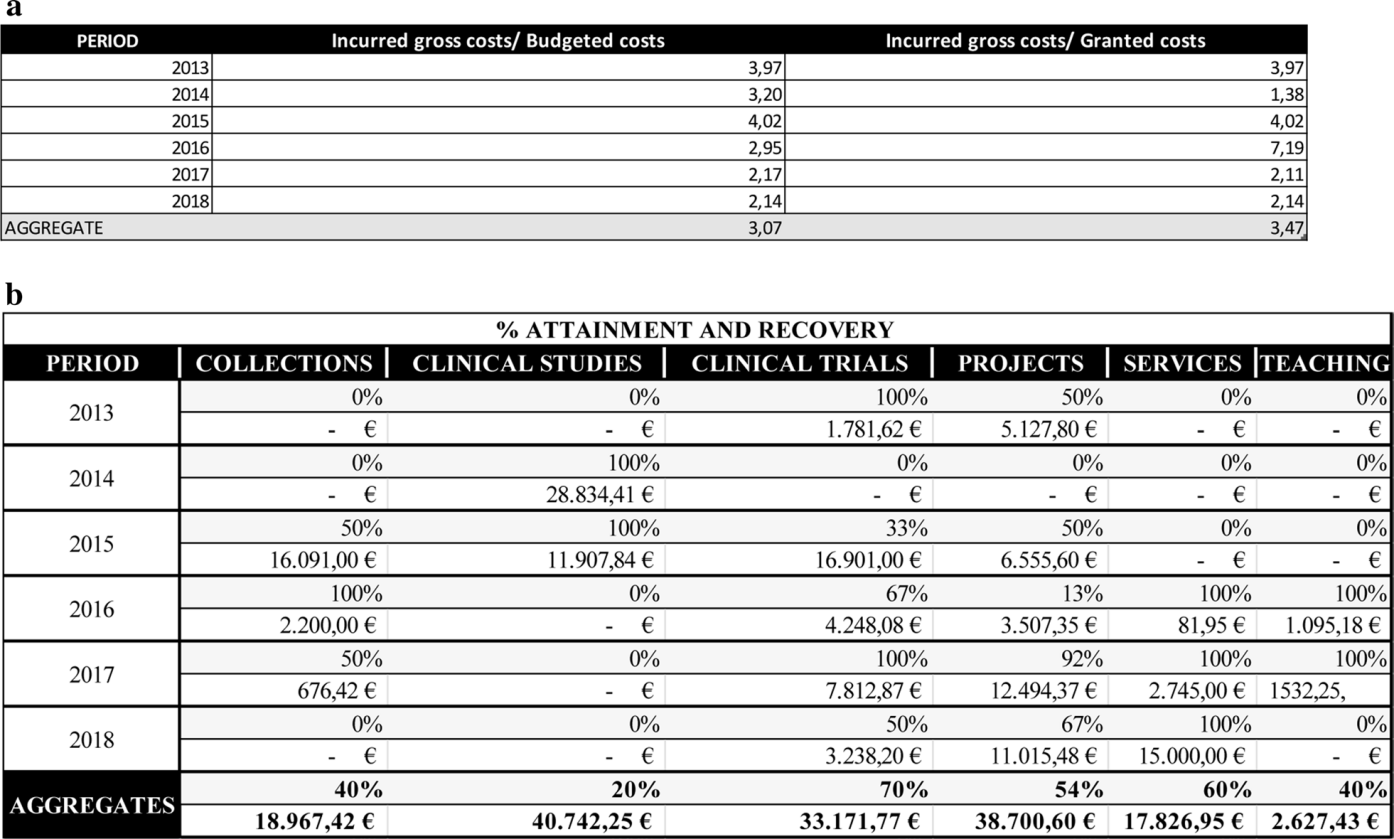
**a** Ratios of estimated recovery and real recovery of the investment of Spanish HIV HGM BioBank. 2013–2018. **b** Costs recovery policy by type of activity of the Spanish HIV HGM BioBank, 2013–2018

The annual monitoring of ratios between IGC, BC and GDC gives information on the investments made since the implementation of the CRP. Sequential monitoring sets out financial objectives in terms of acceptable and optimal quantities projected for the short and medium term (Fig. 7a). The Spanish HIV HGM BioBank, on average value, invests 3.07 euros per euro budgeted and contributes 3.47 euros per euro recovered.

As it has been stated before, a provisional monitoring of this ratio by means of control charts is useful within the limits of acceptance of an economic counter offer. To achieve this objective, Spanish HIV HGM BioBank uses a Levey–Jennings control chart starting from IGC/BC ratio (Fig. 6b).

#### Analysis of the costs recovery policy by type of activity

The activity categories are:

- Collection: to create new collections of samples, under whatever level of access, obtained expressly for a particular client/researcher’s request.
- Clinical study: to cooperate with clinical studies financed by public entities that require prospective samples for the applicant’s exclusive use.
- Teaching: to arrange teaching activities, including theoretical, practical, day courses, etc.
- Project: to work jointly with research projects, by use of retrospective samples.
- Service: to work in laboratory technical services, advising, quality control, etc.
- Clinical Trial: to work jointly with clinical trials.

Knowing why certain of the Spanish HIV HGM BioBank’s routes and activities attract fewer or larger quantities of funds sheds light on the elements of the operation that work remarkably well and those that need to be improved. Management can use this to design future strategies by increasing the range of products and services, and/or directing its efforts to optimize the ones with particular relevance, with the goal of unbeatable excellence (Fig. 7b).

The main paths to recover the investment, in net volume, are clinical studies and clinical trials as they are based on stable financing, followed by the transfer of retrospective samples. Analyzing the percentage of requests accepted, the number of budgets approved is high. This is remarkable because the implementation of the CRP in sample transfer deals could negatively impact in clients, who are used to request free-of-charge biological material. These points that the dissemination strategy has been well conducted and constantly improved, leading us to expect upward trending predictions.

## Discussion

The Spanish HIV HGM BioBank’s CRP goal is to define (1) costs for each task, (2) strategy of recovery and (3) set up a scale of discounts. The scope of this model is applicable in all the actions aimed to the achievement of the entity’s sustainability, accountable management and financial projections. This platform is able to know its minimum investment needs in a certain period of time since this policy was initiated. Though these data do not determine an unequivocal amount of critical resources for a future period, they allow to measure reference margins and develop preventive actions to assure the availability of economic means. Coming ahead of cutbacks, scarcity of resources, equipment acquisition and implementation of new services or prospective samples collections is essential to not endanger the future of the BioBanks [14–17].

Costs analysis based in ABC model permitted to detect which expenditure items are particularly sensitive [18, 19]. This information gives the chance of prioritise the expenditure in those projects with a higher scientific impact and relevant applications.

This is a crucial study, given that the Spanish HIV HGM BioBank activities can entail an amount of costs that exceeds the BioBank’s resources, compromising its stability. Moreover, applicants can frequently only pay once the study is ended or the funding entities grant the money. This fact results in a period of moths/years where the BioBank has to bear a sustained investment without any recovery. In this sense, a Levey–Jennings analysis allows to have an initial control over any investment, considering that Spanish HIV HGM BioBank is performing a capital invest of 3.5 times over the recovery, far from desirable 0 rate [20].

Lack of knowledge about BioBank needs and operational procedures among researches is another important hurdle that must be overcome in the development of effective CRPs [14–16]. In the past, clients obtained biomaterial at zero cost. As a consequence, the requests of payment for a traditionally free service generates misgivings and many problems in the budget signing. This must be solved by transparency and communication from BioBanks. A proper spread of CRP allowed the researches to be aware of the importance of adding BioBank costs in their request to funding bodies, in order to avoid the not fulfilment of the study and/or the survival of BioBanks.

Another positive fact of this policy is raising awareness of the value of biomaterial [17, 18]. The volume of requested sample must be the minimum necessary because they are finite resources. Instead, when the material was free, this volume was increased in case of unexpected events, and samples were spoiled. This situation is reversed if the samples have a price. In addition, CRP let customers know the real costs of the BioBank activities plus the low fraction or recovery requested, obtaining a transparent and clear communication of its spirit of service and its effort in the advance of knowledge.

Among the current challenges, the heterogeneous essence of this platforms make it difficult to determine a unique model or economic framework. Moreover, there is not specific software for BioBanks’ sustainability and sometimes accounting data is short or absent. Currently, the best path to develop economic policies is the study of previous experiences and contributions among BioBanks.

Nowadays, self-finance is utopic in the short-term, but Spanish HIV HGM BioBank is achieving a recovery of part of its costs. This feedback allows to improve the management of future needs in equipment acquisition, collections, prospective studies or clinical trials with a greater leverage of the BioBanks capital.

## Conclusions

A business model to maximize BioBank usage for seeking sustainable development and operation.

A sustainability costs recovery policy (CRP) for financial control, including fixed and variable costs, has been developed for the BioBank.

An economic plan allowing future projects including rules for acceptance or rejection, internal investment limits, minimum recovery needs in short medium term, deviation detection system and a register of capital recovery by period and type of service has been developed by our BioBank.

Our model can help BioBanks that do not have a costs recovery model to design it, as well as to detect improvements and functional revisions by those experienced in this field.

The future may be unclear, but what is certain is that sustainability is an issue that needs to be resolved to enable BioBanking to continue.

## Limitations

The future may be unclear, but what is certain is that sustainability is an issue that needs to be resolved to enable BioBanking to continue.

## Supplementary information

**Additional file 1.** List of papers directly or indirectly generated by the Spanish HIV HGM BioBank.

## Data Availability

All relevant data are within the paper.

## Abbreviations

HIV: human immunodeficiency virus
HGM: Hospital Gregorio Marañón
CRM: costs recovery model
RCP: recovery cost policy
RED RIS: Spanish AIDS Research Network
GC: gross costs
IGC: incurred gross costs
BC: budgeted costs
GDC: granted costs
CRP: costs recovery policy
