# Supplementary material for: Assessing and measuring financial sustainability model of the Spanish HIV HGM BioBank
Source: J Transl Med. 2020 Jan 6;18:6. doi: 10.1186/s12967-019-02187-w (PMC6943905; doi:10.1186/s12967-019-02187-w)
Supplement: Supplementary file 1 — Additional file 1. List of papers directly or indirectly generated by the Spanish HIV HGM BioBank. [file 12967_2019_2187_MOESM1_ESM.pdf]

Title	Authors	Review	Year	IF	Quartile / Decile
DC-SIGN ligation on dendritic cells results in ERK and PI3K activation and modulates cytokine production.	Caparrós E, Muñoz P., Sierra-Filardi E, Serrano-Gómez D, Puig-Kröger A, Ridríguez-Fernández JL, Mellado M, Sancho J, Zubiaur M, Corbí AL.	Blood, 107:3950-3958, 2006.	2006	10,370	(1st Decilee)
RUNX3 negatively regulates CD36 expression in monocyte-derived dendritic cells and alternatively activated macrophages.	Puig-Kröger, A., Serrano-Gómez, D., Martínez-Muñoz, L., Domínguez-Soto, A., Ruiz-Velasco, N., Fernández-Ruiz, E., Groner, Y., Tandon, N., Corbí, A.L*., y Vega, M.A*	J. Immunol. 177:2107-2114. 2006.	2006	5,520	(1st Quartile)
Analysis of DC-SIGN (CD209)Functional Variants in Patients with Tuberculosis.	Gómez, L.M., Anaya, J-M., Sierra-Filardi, E., Cadena, J., Corbí A.L., y Martín, J. Analysis of DC-SIGN (CD209)	Human Immunol. 67:808–811, 2006.	2006	2,605	(2nd Quartile)
The DC-SIGN-related lectin LSECtin mediates antigen capture and pathogen binding by human myeloid cells	Dominguez-Soto A, Aragoneses-Fenoll L, Martin-Gayo E, Martinez-Prats L, Colmenares M, Naranjo-Gomez M, Borras FE, Munoz P, Zubiaur M, Toribio ML, Delgado R, Corbi AL.	Blood, Jun 15;109(12):5337-5345. 2007	2007	10,896	(1st Decilee).
Inmunoferon® modulates dendritic cell pathogen-recognition capabilities by targeting DC-SIGN.	Serrano-Gómez, D., Martínez-Nuñez, RT., Sierra-Filardi, E., Colmenares, M., Pla, J., Rivas, L., Jimenez-Barbero, J., Alonso-Lebrero, JA., González, S., y Corbí, AL.	Antimicrob Agents Chemother, 51:2313-2323, 2007.	2007	4,390	(1st Quartile)
Spanish cohort of naïve HIV-infected patients (CoRIS): rationale, organization and initial results	Caro-Murillo AM, Castilla J, Pérez-Hoyos S, Miró JM, Podzamczer D, Rubio R, Riera M, Viciana P, López Aldeguer J, Iribarren JA, de los Santos-Gil I, Gómez-Sirvent JL, Berenguer J, Gutiérrez F, Saumoy M, Segura F, Soriano V, Peña A, Pulido F, Oteo JA, Leal M, Casabona J, del Amo J, Moreno S; Grupo de trabajo de la Cohorte de la Red de Investigación en Sida (CoRIS)	Enferm Infecc Microbiol Clin. 2007 Jan;25(1):23-31. Spanish.	2007	1,096	> 2º Quartile
No Major Differences in the Functional Profile of HIV Gag and Nef-Specific CD8 Reponses between Long-Term Nonprogressors and Typical Progressors	Mariola López, Vincent Soriano, Sara Lozano, Celia Ballesteros, Almudena Cascajero, Berta Rodés, Elvira De La Vega, Juan González-Lahoz, and José M. Benito	AIDS Res Hum Retroviruses. 2008 Sep;24(9):1185-95.	2008	2,024	(2nd Quartile)
AM3, a natural glycoconjugate, induces the functional maturation of human dendritic cells.	Martín-Vilchez S, Molina-Jiménez F, Alonso-Lebrero JL, Sanz-Cameno P, Rodríguez-Muñoz Y, Benedicto I, Roda-Navarro P, Trapero M, Aragoneses-Fenoll L, González S, Pivel JP, Corbí AL, López-Cabrera M, Moreno-Otero R, Majano PL.	Br. J. Pharmacol. 154:698-708, 2008.	2008	4,838	(1st Quartile)
Structural requirements for multimerization of the pathogen receptor dendritic cell-specific ICAM3-grabbing non-integrin (CD209) on the cell surface.	Serrano-Gómez D, Sierra-Filardi E, Martínez-Nuñez RT, Caparrós E, Delgado R, Muñoz-Fernández MA, Abad MA, Jimenez-Barbero J, Leal M, Corbí AL.	J Biol Chem. 2008 Feb 15;283(7):3889-3903.	2008	5,520	(1st Quartile)
The pathogen receptor liver and lymph node sinusoidal endotelial cell C-type lectin is expressed in human Kupffer cells and regulated by PU.1.	Domínguez-Soto, A., Aragoneses-Fenoll, L., Corcuera, M., Miquilena-Colina, M.E., García-Monzón, C., Gömez-Aguado, F., Bustos, M., Corbí A.L.	Hepatology, 49:287-296, 2009.	2009	10,840	(1st Decilee).
HIV infection in immigrants in Spain: Epidemiological characteristics and clinical presentation in the CoRIS Cohort (2004-2006)".	Caro-Murillo AM, Gutiérrez F, Manuel Ramos J, Sobrino P, Miró JM, López-Cortés LF, Tural C, Moreno A, de Los Santos Gil I, Murillas Angoiti J, Camino X, Salavert Lletí M, Rubio R, Moreno S, Del Amo J; CoRIS	Enferm Infecc Microbiol Clin. 2009 May;27(7):380-8.	2009	1,482	> 2º Quartile
Correlation between the Trofile test and virological response to a short-term maraviroc exposure in HIV-infected patients	Genebat M, Ruiz-Mateos E, León JA, González-Serna A, Pulido I, Rivas I, Ferrando-Martínez S, Sánchez B, Muñoz-Fernández MA, Leal M	J Antimicrob Chemother. 2009 Oct;64(4):845-9.	2009	4,352	(1st Quartile)
Non-steroidal anti-inflammatory drugs increase the antiretroviral activity of nucleoside reverse transcriptase inhibitors in HIV type-1-infected T-lymphocytes: role of multidrug resistance protein 4	Clemente MI, Alvarez S, Serramía MJ, Turriziani O, Genebat M, Leal M, Fresno M, Muñoz-Fernández MA	Antivir Ther. 2009;14(8):1101-11.	2009	4,322	(1st Quartile)
ZNRD1 (Zinc Ribbon Domain–Containing 1) Is a Host Cellular Factor That Influences HIV-1 Replication and Disease Progression	Ester Ballana, Jordi Senserrich, Eduardo Pauls, Rosa Faner, Josep Maria Mercader, Frederic Uyttebroeck, Eduard Palou, Maria Pau Mena, Eulalia Grau, Bonaventura Clotet, Lidia Ruiz, Amalio Telenti, Angela Ciuffi, and José A. Este	Clin Infect Dis. 2010 Apr 1;50(7):1022-32.	2010	9,154	(1st Decilee).
Sensitivity of seven HIV subtyping tools differs among subtypes/recombinants in the SpanishcohortofnaïveHIV-infectedpatients(CoRIS)	Gonzalo Yebraa , MigueldeMuldera , LeticiaMartína , SantiagoPérez-Cachafeirob , CarmenRodríguezc ,Pablo Labargad, FedericoGarcíae, CristinaTuralf, ÀngelsJaéng, GemmaNavarroh, Á. Holguín,∗ on behalf of the Cohort of the Spanish AIDS Research Network (CoRIS)	Antiviral Research xxx (2010) xxx–xxx. GModel AVR-2728; No.ofPages7	2010	4,031	(1st Quartile)
Long-Term Efficacy and Safety of Fosamprenavir in Human Immunodeficiency Virus-Infected Pediatric Patients	Palladino, Claudia PhD; Briz, Verónica PhD; Policarpo, Sergio Negre MD; Silveira, Laura Fernandez MD; de José, M Isabel MD; González-Tomé, M Isabel MD; Moreno, David MD; León Leal, Juan A. MD; Mellado, M José MD; de Ory, Santiago J. BSc; Ramos, José T. MD; Muñoz-Fernández, M Ángeles PhD, MD	Pediatr Infect Dis J. 2010 Jun;29(6):563-6.	2010	3,064	(1st Decilee)

Evaluation of the Effect of Enfuvirtide in 11 HIV-1 Vertically Infected Pediatric Patients Outside Clinical Trials	Palladino C, Briz V, González-Tomé MI, León Leal JA, Navarro ML, de José MI, Ramos JT, Muñoz-Fernández MA	AIDS Res Hum Retroviruses. 2010 Mar; 26(3):301-5.	2010	2,082	> 2º Quartile
Sensitivity of seven HIV subtyping tools differs among subtypes/recombinants in the Spanish cohort of naïve HIV-infected patients (CoRIS).	Yebra G, de Mulder M, Martín L, Pérez-Cachafeiro S, Rodríguez C, Labarga P, García F, Tural C, Jaén A, Navarro G, Holguín A; Cohort of Spanish AIDS Research Network (CoRIS)	Antiviral Res. 2011 Jan;89(1):19-25. Epub 2010 Nov 9.	2010	4,031	(1st Quartile)
Adipokine profiles and lipodystrophy in HIV-infected children during the first 4 years on highly active antiretroviral therapy	Resino S, Micheloud D, Lorente R, Bellón JM, Navarro ML, Muñoz-Fernández MA	HIV Med. 2010 May 17	2010	3,064	(1st Decilee)
"Gene Therapy in HIV-Infected Cells to Decrease Viral Impact by Using an Alternative Delivery Method".	Gonzalo T, Clemente MI, Chonco L, Weber ND, Díaz L, Serramía MJ, Gras R, Ortega P, de la Mata FJ, Gómez R, Lopez-Fernández LA, Muñoz-Fernández MA, Jiménez JL.	ChemMedChem. 2010; 5(6):921-9.	2010	3,306	(1st Quartile)
"Phenotype and functional analysis of human monocytes-derived dendritic cells loaded with a carbosilane dendrímer"	Pion M, Serramia MJ, Diaz L, Bryszewska M, Gallart T, García F, Gómez R, de la Mata FJ, Muñoz-Fernandez MA	Biomaterials. 2010; 31(33):8749-58.	2010	4,630	(1st Decilee)
Association between lipodystrophy and leptin in human immunodeficiency virus-1-infected children receiving lopinavir/ritonavir-based therapy.	Resino S, Palladino C, Lorente R, Micheloud D, Bellón JM, Larru B, Gutiérrez MD, de José MI, Polo R, Muñoz-Fernández MA; Spanish Group of Pediatric HIV Infection.	Pediatr Infect Dis J. 2010 Aug;29(8):774-7.	2010	3,064	(1st Decilee)
Hepatitis C virus replication in Caucasian HIV controllers	E. Ruiz-Mateos, K. Machmach, M. C. Romero-Sanchez, S. Ferrando-Martinez, P. Viciana,M. Del Val,M. A. Munoz-Fernandez, M. Genebat and M. Leal on behalf of the Cohort of the Spanish AIDS Research Network (CoRIS)	Journal of Viral Hepatitis, 2011, 18, e350–e357.	2011	4,088	(1st Quartile)
HBV primary drug resistance in newly diagnosed HIV-HBV-coinfected individuals in Spain.	Tuma P, Pineda JA, Labarga P, Vidal F, Rodriguez C, Poveda E, Santos J, Gonzalez-García J, Sobrino P, Tural C, Soriano V; CoRIS Study Group	Antivir Ther. 2011;16(4):585-9	2011	3,161	(2nd Quartile)
Association between IL28B gene polymorphisms and plasma HCV-RNA levels in HIV/HCV-co-infected patients	Labarga P, Soriano V, Caruz A, Poveda E, Di Lello F, Hernandez-Quero J, Moreno S, Bernal E, Miró JM, Leal M, Gutierrez F, Portilla J, Pineda JA	AIDS 2011Mar 27;25(6):761-6. doi: 10.1097/QAD.0b013e32834488e7	2011	6,245	(1st Decilee).
First-line antiretroviral therapy with a protease inhibitor versus non-nucleoside reverse transcriptase inhibitor and switch at higher versus low viral load in HIV-infected children: an open-label, randomised phase 2/3 trial".	The PENPACT-1 (PENTA 9/PACTG 390) Study Team	Lancet Infect Dis. 2011 Jan 31.	2011	18,095	(1st Decilee)
The role of a bioresource research impact factor as an incentive to share human resources.	Anne Cambon-Thomsen, Gudmundur A Thorisson, & Laurence Mabile for the BRIF workshop group	Nature Genetics.2011, June; 42 (6).	2011	34,520	(1st Decilee)
Incidence and Clinical Characteristics of Spontaneous Hepatitis C Virus (HCV) Clearance After Liver Transplantation (LT) in HIV/HCV-Coinfected Patients: Results of the FIPSE OLT-HIV-Cohort Study (2002-11).	José M. Miró, Christian Manzardo, Jesús Fortún, Elisa Cordero, Santos del Campo, Susanna Naggie, Christian Brander, Isabel García-Merino, Asuncion Moreno, Antonio Rimola, and the Spanish OLT in HIV-Infected Patients Working Group.	CROI	2011	NO TIENE FACTOR DE IMPACTO	
Imputation of the Date of HIV Seroconversion in a Cohort of Seroprevalent Subjects: Implications for Analysis of Late HIV Diagnosis	Sobrino-Vegas P, Pérez-Hoyos S, Geskus R, Padilla B, Segura F, Rubio R, Del Romero J, Santos J, Moreno S, Del Amo J	AIDS Res Treat. 2012;2012:725412. Epub 2011 Oct 15.	2011	2,246	> 2º Quartile
Intensification of Antiretroviral Therapy with a CCR5 Antagonist in Patients with Chronic HIV-1 Infection: Effect on T Cells Latently Infected	Carolina Gutiérrez, Laura Díaz, Alejandro Vallejo, Beatriz Hernández-Novoaa, María Abad, Nadia Madrid, Viktor Dahl, Rafael Rubio, Ana M. Moreno, Fernando Dronda, Jose´ Luis Casado, Enrique Navas, María Jesús Pérez-Elías, Javier Zamora, Sarah Palmer, Eduardo Muñoz, María Ángeles Muñoz-Fernández, Santiago Moreno.	PLoS One 2011; 6(12):e27864	2011	4,092	(1st Quartile)
Sensitivity of seven HIV subtyping tools differs among subtypes/recombinants in the Spanish cohort of naïve HIV-infected patients (CoRIS)	Gonzalo Yebra , Miguel de Mulder, Leticia Martín, Santiago Pérez-Cachafeiro, Carmen Rodríguez, Pablo Labarga, Federico García, Cristina Tural, Àngels Jaén, Gemma Navarro, Á. Holguín, on behalf of the Cohort of the Spanish AIDS Research Network (CoRIS)	Antiviral Res 89(1):19-25	2011	3,925	(1st Quartile)
Carbosilane Dendrimer 2G-NN16 Represses Tc17 Differentiation in primary T CD8+ Lymphocytes	Rafael Gras, María I. García, Rafael Gońez, F. Javier de la Mata, M. Angeles Muñoz-Fernández, and Luís A. López-Fernández	Mol Pharm. 2012 Jan 1;9(1):102-10. Epub 2011 Nov 16.	2011	4,750	(1st Quartile)

Safety and immunogenicity of a modified pox vector-based HIV/AIDS vaccine candidate expressing Env, Gag, Pol and Nef proteins of HIV-1 subtype B (MVA-B)in healthy HIV-1-uninfected volunteers: A phase I clinical trial (RISVAC02)	Felipe García, Juan Carlos López Bernaldo de Quirós, Carmen E. Gómez, Beatriz Perdiguero, Jose L. Nájera, Victoria Jiménez, Juan García-Arriaza, Alberto C. Guardo, Iñaki Pérez, Vicens Díaz-Brito, Matilde Sánchez Conde, Nuria González, Amparo Alvarez, José Alcamí, José Luis Jiménez, Judit Pich, Joan Albert Arnaiz, María J. Maleno, Agathe León, María Angeles Muñoz-Fernández, Peter Liljeström, Jonathan Weber, Giuseppe Pantaleo, José M. Gatell, Montserrat Plana, Mariano Esteban	Vaccine. 2011 Oct 26;29(46):8309-16. Epub 2011 Sep 9.	2011	3,766	(1st Quartile)
Estudio preliminar sobre las alteraciones fenotípicas de las células Treg causadas por la infección VIH en pacientes adultos infectados	Jaramillo-Ruiz LD, Muñoz-Fernández MA, Correa R	Revista Complutense de Ciencias Veterinarias. 2011, 5(2):49-64	2011	NO TIENE FACTOR DE IMPACTO	
Adipokine profiles and lipodystrophy in HIV-infected children during the first 4 years on highly active antiretroviral therapy	S Resino, D Micheloud, R Lorente, JMa Belló n, MaL Navarro and MaA Muñoz-Fernández	HIV Medicine (2011), 12, 54–60	2011	3,006	(2nd Quartile).
The cohort of the spanish hiv research network (CoRIS) and its associated biobank; organizational issues, main findings and losses to follow-up	Sobrino-Vegas P, Gutiérrez F, Berenguer J, Labarga P, García F, Alejos-Ferreras B, Muñoz MA, Moreno S, del Amo J; CoRIS.	Enferm Infec Micro Cl. 2011; 29(9): 645-53.	2011	1,491	> 2º Quartile
Virological response after a short-term CCR5 antagonist exposure in HIV-infected patients: Frequency of subjects with virological response and associated factors	Ruiz-Mateos E, González-Serna A, Genebat M, Machmach K, Vidal F, Muñoz-Fernández A, Ferrando-Martinez S, Leal M.	Antimicrob Agents Ch	2011	4,841	(1st Decilee)
Cardiovascular risk markers are increased in HIV-infected children with lipodystrophy syndrome	Guzmán-Fulgencio M, Micheloud D, Lorente R, Bellón JM, Gomez MI, Gurbindo MD, León JA, Muñoz-Fernández MÁ, Resino S.	J Infect. 2011 Mar;62(3):240-3. Epub 2011 Jan 21.	2011	4,126	(1st Quartile)
Causes of death in pediatric patients vertically infected by the human immunodeficiency virus type 1 in Madrid, Spain, from 1982 to mid-2009".	Palladino C, Climent FJ, José MI, Jimenez De Ory S, Bellón JM, Guillén S, Gurbindo MD, González-Tomé I, Mellado MJ, Martínez Pérez J, Calvo C, Ramos JT, Muñoz-Fernández MA.	Pediatr Infect Dis J. 2011 Jun;30(6):495-500.	2011	3,577	(1st Decilee)
Etravirine-based highly active antiretroviral therapy in HIV-1-infected paediatric patients	Briz V, Palladino C, Navarro M, Jiménez de Ory S, González-Tomé M, León J, Núñez-Cuadros E, de José M, Ramos J, Muñoz-Fernández M	HIV Med. 2011 Aug;12(7):442-446. doi: 10.1111/j.1468-1293.2010.00907.x. Epub 2011 Mar 13.	2011	3,006	(2nd Quartile)
Hepatitis C virus replication in Caucasian HIV controllers	Ruiz-Mateos E, Machmach K, Romero-Sanchez MC, Ferrando-Martinez S, Viciana P, Del Val M, Muñoz-Fernandez M A , Genebat M and Leal M on behalf of the Cohort of the Spanish AIDS Research Network (CoRIS).	Viral Hepatitis. 2011; 18(7):350-7. J	2011	4,088	(1st Quartile)
Drug resistance prevalence and HIV-1 variant characterization in the naive and pretreated HIV-1-infected paediatric population in Madrid, Spain	de Mulder M, Yebra G, Martín L, Prieto L, Mellado MJ, Rojo P, Muñoz-Fernández MÁ, Jiménez de Ory S, Ramos JT, Holguín A; Madrid cohort of HIV-infected children	J Antimicrob Chemother. 2011 Oct;66(10):2362-71	2011	5,068	(1st Decilee)
PEGylated poly(ethylene imine) copolymer-delivered siRNA inhibits HIV replication in vitro	Nick D. Weber, Olivia M. Merkel, Thomas Kissel , María Ángeles Muñoz-Fernández	J Control Release. 2012 Jan 10;157(1):55-63	2011	7,633	(1st Decilee)
Differential prevalence of the HLA-C −35 CC genotype among viremic long term non-progressor and elite controller HIV+ individuals.	Ballana E, Ruiz-de Andres A, Mothe B, Ramirez de Arellano E, Aguilar F, Badia R, Grau E, Clotet B, del Val M, Brander C, Esté JA	Immunobiology. 2012 Sep;217(9):889-94. Epub 2012 Jan 8.	2012	2,814	> 2º Quartile
Rare LEDGF/p75 genetic variants in white long-term nonprogressor HIV+ individuals.	Ballana E, Gonzalo E, Grau E, Iribarren JA, Clotet B, Este JA.	AIDS. 2012 Feb 20;26(4):527-8.	2012	6,264	(1st Decilee)
Characterization of LEDGF/p75 genetic variants and association with HIV-1 disease progression	Messiaen P, De Spiegelaere W, Alcami J, Vervisch K, Van Acker P, Verhasselt B, Meuwissen P, Calonge E, Gonzalez N, Gutierrez-Rodero F, Rodriguez-Martín C, Sermijn E, Poppe B, Vogelaers D, Verhofstede C, Vandekerckhove L.	PLoS One. 2012;7(11):e50204	2012	3,730	(1st Quartile).
Outcome of HCV/HIV-coinfected liver transplant recipients: a prospective and multicenter cohort study	Miro JM, Montejo M, Castells L, Rafecas A, Moreno S, Agüero F, Abradelo M, Miralles P, Torre-Cisneros J, Pedreira JD, Cordero E, de la Rosa G, Moyano B, Moreno A, Perez I, Rimola A; Spanish OLT in HIV-Infected Patients Working Group investigators	Am J Transplant. 2012 Jul;12(7):1866-76. doi: 10.1111/j.1600-6143.2012.04028.x. Epub 2012 Apr 4.	2012	6,192	(1st Quartile)
Vector replication and expression of HIV-1 antigens by the HIV/AIDS Vaccine Candidate MVA-B is not affected by HIV-1 protease inhibitors	García-Arriaza J, Arnáez P, Jiménez JL, Gómez CE, Muñoz-Fernández MA, Esteban M.	Virus Res 2012; 167(2): 391-396.	2012	2,745	(2nd Quartile)
HIV-1 tropism evolution after short-term maraviroc monotherapy in HIV-1-infected patients	Gonzalez-Serna A, Romero-Sánchez MC, Ferrando-Martinez S, Genebat M, Vidal F, Muñoz-Fernández MA, Abad MA, Leal M, Ruiz-Mateos E.	Antimicrob Agents Ch 2012; 56(7):3981-3.	2012	4,565	(1st Decilee)
Differential gag-specific polyfunctional T cell maturation 2 patterns in HIV-1 Elite Controllers	Ferrando-Martínez S, Casazza JP, Leal M, Machmach K, Muñoz-Fernández MÁ, Viciana P, Koup RA, Ruiz-Mateos E.	J Virol. 2012 Apr;86(7):3667-74	2012	5,076	(1st Quartile)
Plasmacytoid Dendritic Cells Reduce HIV Production in Elite Controllers	Machmach K, Leal M, Gras C, Viciana P, Genebat M, Franco E, Boufassa F, Lambotte O, Herbeuval JP, Ruiz-Mateos E.	J Virol. 2012 Apr;86(8):4245-52	2012	5,076	(1st Quartile)

Differential alterations of the CD4 and CD8 T cell subsets in HIV-infected patients on highly active antiretroviral therapy with low CD4 T cell restoration	Gema Méndez-Lagares, Antonio García-Pergañeda, Marí del Mar del Pozo-Balado, Miguel Genebat,Ezequiel Ruiz-Mateos, María García García, María Ángeles Muñoz-Fernández, Yolanda María Pacheco and Manuel Leal.	J Antimicrob Chemoth. 2012, 66(3):645-9	2012	5,338	(1st Decilee)
Most HIV type 1 non-B infections in the Spanish cohort of antiretroviral treatment-naïve HIV-infected patients (CoRIS) are due to recombinant viruses	Yebra G, de Mulder M, Martín L, Rodríguez C, Labarga P, Viciana I, Berenguer J, Alemán MR, Pineda JA, García F, Holguín A; Cohort of the Spanish AIDS Research Network (CoRIS)	J Clin Microbiol. 2012 Feb;50(2):407-13	2012	4,068	(1st Quartile)
The inhibition of Th17 immune response in vitro and in vivo by the carbosilane dendrimer 2G-NN16	Gras R, Relloso M, García MI, de la Mata FJ, Gómez R, López-Fernández LA, Muñoz-Fernández MA	Biomaterials. 2012 May;33(15):4002-9	2012	7,604	(1st Decilee)
Vector replication and expression of HIV-1 antigens by the HIV/AIDS vaccine candidate MVA-B is not affected by HIV-1 protease inhibitors.	García-Arriaza J, Arnáez P, Jiménez JL, Gómez CE, Muñoz-Fernández MÁ, Esteban M	Virus Res. 2012 Aug;167(2):391-6. Epub 2012 May 30.	2012	2,754	(2nd Quartile)
Direct phenotypical and functional dysregulation of primary human B cells by human immunodeficiency virus (HIV) type 1 in vitro	Perisé-Barrios AJ, Muñoz-Fernandez MÁ, Pion M	PLoS One. 2012;7(7):e39472	2012	3,730	(1st Quartile)
Trends in Drug Resistance Prevalence in HIV-1 Infected Children in Madrid: 1993-2010 Analysis	de Mulder M, Yebra G, Navas A, Martín L, de Jose MI, Navarro ML, de Ory SJ, Gonzalez-Granado I, Mellado MJ, Ramos JT, Holguin A	Pediatr Infect Dis J. 2012 Jul 10.	2012	3,569	(1st Decilee)
In Vitro Studies of Water-Stable Cationic Carbosilane Dendrimers as Delivery Vehicles for Gene Therapy against HIV and Hepatocarcinoma	de Las Cuevas N, García-Gallego S, Rasines B, de la Mata FJ, Guijarro LG, Muñoz-Fernández MA, Gómez R	Curr Med Chem. 2012 Sep 3	2012	4,070	(1st Decilee)
High Drug Resistance Prevalence among Vertically HIVInfected Patients Transferred from Pediatric Care to Adult Units in Spain	Miguel de Mulder, Gonzalo Yebra, Adriana Navas, María Isabel de José, María Dolores Gurbindo, María Isabel González-Tomé, María José Mellado, Jesús Saavedra-Lozano, María Ángeles Muñoz-Fernández, Santiago Jiménez de Ory, José Tomás Ramos, África Holguín	PLoS One 2012; 7(12):e52155	2012	3,730	(1st Quartile)
Potent and sustained antiviral response of raltegravir-based highly active antiretroviral therapy in Human Immunodeficiency Virus Type 1 infected children and adolescents	Briz V, León-Leal JA, Palladino C, Moreno-Perez D, de Ory SJ, De José MI, González-Tomé MI, Martín CG, Pocheville I, Ramos JT, Leal M, Muñoz-Fernández MÁ.	Pediatr Infect Dis J. 2012 Mar;31(3):273-7.	2012	3,569	(1st Decilee)
Preterm neonates show marked leukopenia and lymphopenia that are associated with increased regulatory T-cell values and diminished IL-7	Correa-Rocha R, Pérez A, Lorente R, Ferrando-Martínez S, Leal M, Gurbindo D, Muñoz-Fernández MÁ	Pediatr Res. 2012 May;71(5):590-7	2012	2,673	(1st Quartile)
Detectable viral load aggravates immunosenescence features of CD8 T-cell subsets in vertically HIV-infected children	Díaz L, Méndez-Lagares G, Correa-Rocha R, Pacheco YM, Ferrando-Martínez S, Ruiz-Mateos E, del Mar del Pozo-Balado M, León JA, Gurbindo MD, Isabel de José M, Leal M, Muñoz-Fernández MÁ	J Acquir Immune Defic Syndr. 2012 Aug 15;60(5):447-54.	2012	4,653	(1st Quartile)
Carbosilane dendrimer nanotechnology outlines of the broad HIV blocker profile	Chonco L, Pion M, Vacas E, Rasines B, Maly M, Serramía MJ, López-Fernández L, De la Mata J, Alvarez S, Gómez R, Muñoz-Fernández MA	J Control Release. 2012 Aug 10;161(3):949-58	2012	7,633	(1st Decilee)
Specific patterns of CD4-associated immunosenescence in vertically HIV-infected subjects	Méndez-Lagares G, Díaz L, Correa-Rocha R, León Leal JA, Ferrando-Martínez S, Ruiz-Mateos E, Pozo-Balado MM, Gurbindo MD, de José MI, Muñoz-Fernández MA, Leal M, Pacheco YM.	Clin Microbiol Infect 2013 Jun	2012	5,197	(1st Decilee)
Patients on a combined antiretroviral therapy after maraviroc clinical test show no immunovirological impairment	Genebat M, Pulido I, Romero-Sánchez MC, González-Serna A, Ferrando-Martínez S, Machmach K, Pacheco YM, Muñoz-Fernández MÁ, Ruiz-Mateos E, Leal M	Antiviral Res. 2012 Sep;95(3):207-11.	2012	3,925	(1st Quartile)
High Prevalence of X4/DM-Tropic Variants in Children and Adolescents Infected With HIV-1 by Vertical Transmission	Briz V, García D, Méndez-Lagares G, Ruiz-Mateos E, de Mulder M, Moreno-Pérez D, Navarro ML, León-Leal JA, de José MI, Ramos JT, Mellado MJ, González-Tomé MI, Leal M, Muñoz-Fernández MA.	Pediatr Infect Dis J. 2012 Oct;31(10):1048-1052.	2012	3,569	(1st Decilee)
Validation of a Generation 4 Phosphorus-Containing Polycationic Dendrimer for Gene Delivery against HIV-1	Briz V, Serramía MJ, Madrid R, Hameau A, Caminade AM, Majoral JP, Muñoz-Fernández MA.	Curr Med Chem 2012; 19 (29): 5044-5051 (8)	2012	4,070	(1st Decilee)
IL28B Single-Nucleotide Polymorphism rs12979860 Is Associated With spontaneous HIV Control in White Subjects.	K. Machmach, C. Abad-Molina, M. C. Romero-Sanchez,M. A. Abad, S. Ferrando-Martinez, M. Genebat, I. Pulido, P. Viciana, M.F. González-Escribano,M. Del Val,E. Ruiz-Mateos and the HIV Controllers Consortium of the AIDS Spanish Network	J Infect Dis 2013; 207:651-655	2013	5,848	(1st Decilee).
Inversion of the CD4/CD8 ratio despite antiretrovirals in vertically HIV-infected children and young predicts the activation/exhaustion phenotype in CD8+ T cells	Sainz T, Serrano-Villar S, Diaz L, Ramos JT, Navarro M, Moreno S, Muñoz-Fernández MA.	AIDS. 2013 Feb 21	2013	6, 557	(1st Decilee)

Mitochondrial Haplogroups Are Associated With Clinical Pattern of AIDS Progression in HIV-Infected Patients	María Guzmán-Fulgencio, PhD, José Luis Jiménez, PhD, Mónica García-Álvarez, PhD,José María Bellón, BSc, Amanda Fernández-Rodriguez, PhD, Yolanda Campos, PhD,Carmen Rodríguez, PhD,k Juan González-Garcia, MD, PhD, Melchor Riera, MD, PhD,Pompeyo Viciana, MD, PhD, MªÁngeles Muñoz-Fernández, PhD,and Salvador Resino, PhD.	J Acquir Immune Defic Syndr Volume 63, Number 2, June 1, 2013.	2013	4,390	(1st Quartile).
Mechanisms of abrupt HIV disease progression in a cohort of previous elite and viremic HIV controllers	Muntsa Rocafort, Rocío Bellido, Manel Crespo, Jordi Puig, M. Teresa Orodea, Andres Marco,Lidia Ruiz, Jose Alcamí, Bonaventura Clotet, Roger Paredes, Christian Brander, Beatriz Mothe	AIDS vaccine 2013	2013	6, 557	(1st Decilee)
Prevalence of HIV-1 dual infection in long-term nonprogressor-elite controllers	Pernas M, Casado C, Sandonis V, Arcones C, Rodríguez C, Ruiz-Mateos E, Ramírez de Arellano E, Rallón N, Del Val M, Grau E, López-Vazquez M, Leal M, Del Romero J, López Galíndez C.	Acquir Immune Defic Syndr. 2013 Nov 1;64(3):225-31.	2013	6, 557	(1st Decilee)
Identification of a Cluster of HIV-1 Controllers Infected with Low Replicating Viruses	Concepción Casado, Maria Pernas, Virginia Sandonis, Tamara Alvaro-Cifuentes, Isabel Olivares, Rosa Fuentes, Lorena Martínez-Prats, Eulalia Grau, Lidia Ruiz, Rafael Delgado, Carmen Rodríguez, Jorge del Romero, Cecilio López-Galíndez.	Research Article published 30 Oct 2013.| PLOS ONE 10.1371/journal.pone.0077663	2013	3,543	(1st Quartile).
A new tool for the paediatric HIV research: general data from the Cohort of the Spanish Paediatric HIV Network (CoRISpe)	Ma Isabel de Jose, Santiago Jiménez de Ory, Maria Espiau, Claudia Fortuny, Ma Luisa Navarro, Pere Soler-Palacín, Ma Angeles Muñoz-Fernandez and from the working groups of CoRISpe and HIV HGM BioBank	BMC Infectious Diseases 2013, 13:2	2013	2,561	(2nd Quartile)
Long-Term Suppressive Combined Antiretroviral Treatment Does Not Normalize the Serum Level of Soluble CD14	G. Méndez-Lagares, M. C. Romero-Sánchez, E. Ruiz-Mateos, M. Genebat, S. Ferrando-Martínez, M. Á. Muñoz-Fernández, Y. M. Pacheco, and M. Leal.	Journal of Infectious Diseases Advance Access published February 4, 2013. DOI: 10.1093/infdis/jit025	2013	5,848	(1st Decilee).
A new chimeric protein represses HIV-1 LTR-mediated expression 4 by DNA methylase	Alberto Martinez-Colom, Sandra Lasarte , Alejandra Fernández-Pineda , Miguel Relloso , Angeles Muñoz-Fernández	Antiviral Research xxx (2013) xxx–xxx. http://dx.doi.org/1 0.1016/j.an tiviral.2013.0 4.007	2013	3,784	(1st Quartile).
Glycodendrimers as new tools in the search of effective anti-HIV DC-based immunotherapies	Vacas Córdoba E, Pion M, Rasines B, Filippini D, Komber H, Ionov M, Bryszewska M, Appelhans D, Muñoz- Fernández MA.	Nanomedicine-Nanotechnol 2013 16 Mar	2013	5,824	(1st Decilee)
Synergistic activity of carbosilane dendrimers in combination with maraviroc against HIV in vitro	Córdoba EV, Arnáiz E, De La Mata FJ, Gómez R, Leal M, Pion M, Muñoz-Fernández MA.	AIDS.2013 Apr 26.	2013	6, 557	(1st Decilee)
Mitochondrial haplogroups are associated with clinical pattern of AIDS progression in human immunodeficiency virus infected patients	Guzmán-Fulgencio M, Luis Jiménez J, García-Álvarez M, María Bellón J, Fernández-Rodriguez A, Campos Y, Rodríguez C, González-Garcia J, Riera M, Viciana P, Angeles Muñoz-Fernández M, Resino S; On behalf of CoRIS and the HIV Biobank integrated in the Spanish AIDS Research Network. On behalf of LTNPs Cohort integrated in the Spanish AIDS Research Network. .	JAIDS-J ACQ IMM DEF. 2013 Jun 1.	2013	4,394	(1st Quartile).
Development of sulphated and naphthylsulfonated carbosilane dendrimers as topical microbicides to prevent HIV-1 sexual transmission	Enrique Vacas Córdoba, Eduardo Arnaiz, Miguel Relloso, Carlos Sánchez-Torres, Federico García, Lucía Pérez-Alvarez, Rafael Gómez, Francisco J. de la Mata, Marjorie Pion and M Angeles Muñoz-Fernández.	AIDS 2013, 27:000–000. DOI:10.1097/QAD.0b013e32835f2b7a	2013	6, 557	(1st Decilee)
Synergistic activity profile of carbosilane dendrimer G2-STE16 in combination with other dendrimers and antiretrovirals as topical anti-HIV-1 microbicide	Sepúlveda-Crespo D, Lorente R, Leal M, Gómez R, De La Mata FJ, Jiménez JL, Muñoz-Fernández MA.	Nanomedicine. 2013 Oct 14	2013	5,824	(1st Decilee)
Induction of Treg cells after oral immunotherapy in hen's egg-allergic children	Fuentes-Aparicio V, Alonso-Lebrero E, Zapatero L, Infante S, Lorente R, Muñoz-Fernández MA, Correa-Rocha R	Pediatr Allergy Immunol. 2013 Oct 7	2013	3,859	(1st Decilee)
HIV-Antigens Charged on Phosphorus Dendrimers as Tools for Tolerogenic Dendritic Cells-Based Immunotherapy	Córdoba EV, Bastida H, Pion M, Hameau A, Ionov M, Bryszewska M, Caminade AM, Majoral JP, Muñoz-Fernández MA.	Curr Med Chem. 2013 Nov 28	2013	3,715	(1st Quartile)
Study of cationic carbosilane dendrimers as potential activating stimuli in macrophages	Javier Sánchez-Nieves,ab A. Judith Perisé-Barrios,bc Paula Ortega,ab Ángel L. Corbí,d Ángeles Domínguez-Soto,d M. Ángeles Muñoz-Fernández,*bc Rafael Gómez*ab and F. Javier de la Mata*ab	RSC Adv., 2013,3, 23445-23453.	2013	3,708	(1st Quartile).

ACSM4 Polymorphisms Are Associated With Rapid AIDS Progression in HIV-Infected Patients	María Guzmán-Fulgencio, PhD, José L. Jiménez, PhD, María A. Jiménez-Sousa, PhD, José M. Bellón, PhD, Mónica García-Álvarez, PhD, Vicente Soriano, MD, PhD, Paloma Gijón-Vidaurreta, MD, PhD, Enrique Bernal-Morell, MD, PhD, Pompeyo Viciana, MD, PhD, M. Ángeles Muñoz-Fernández, MD, PhD, and Salvador Resino, PhD.	J Acquir Immune Defic Syndr. 2014 Jan 1;65(1):27-32	2014	4,390	(1st Quartile).
Cardiovascular biomarkers in vertically HIV-infected children without metabolic abnormalities	Sainz T, Diaz L, Navarro ML, Rojo P, Blázquez D, Ramos JT, de José MI, Alvarez-Fuente M, Serrano-Villar S, Mellado MJ, Muñoz-Fernández MA; Madrid Cohort of HIV-infected children and adolescents, integrating the Pediatric branch of the National AIDS Research Network of Spain (CORISPE).	Atherosclerosis. 2014 Jan 23;233(2):410-414. doi: 10.1016/j.atherosclerosis.2014.01.025.	2014	3,994	(1st Quartile)
Functionally defective high density lipoproteins (HDL) are related to heightened T cell activation in vertically HIV-infected adolescents	Sainz T*, Ortega-Hernández A, Navarro ML, Rojo P, Ramos JT, de José MI, Alvarez-Fuente M, Díaz L, Serrano-Villar S, Estrada V, Gomez-Garre D, Mellado MJ, Muñóz-Fernández MA	J Acquir Immune Defic Syndr. 2014 Jul 1;66(3):265-9	2014	4,556	(1st Quartile).
Subclinical Atherosclerosis and Markers of Immune Activation in HIV-Infected Children and Adolescents: The CaroVIH Study	Sainz T*, Navarro ML, Díaz L, Rojo P, Blázquez D, de José MI, Ramos JT, Serrano-Villar S, Martínez J, Medrano C, Muñóz-Fernández MA, Mellado MJ.	J Acquir Immune Defic Syndr. 2014 Jan 1;65(1):42-9.	2014	4,556	(1st Quartile).
The CD4/CD8 ratio as a marker T-cell activation, senescence and activation/exhaustion in treated HIV-infected children and young adults	Sainz T, Serrano-Villar S, Díaz L, González Tomé MI, Gurbindo MD, de José MI, Mellado MJ, Ramos JT, Zamora J, Moreno S, Muñoz-Fernández MA.	AIDS. 2013 Jun 1;27(9):1513-6.	2014	6, 557	(1st Decilee)
Low Thymic Output, Peripheral Homeostasis Deregulation, and Hastened Regulatory T Cells Differentiation in Children with 22q11.2 Deletion Syndrome.	Ferrando-Martínez S, Lorente R, Gurbindo D, De José MI, Leal M, Muñoz-Fernández M, Correa-Rocha R.	J Pediatr. 2014 Apr;164(4):882-9	2014	3,790	(1st Decilee)
Prostaglandin E2 Reduces the Release and Infectivity of New Cell-Free Virions and Cell-To-Cell HIV-1 Transfer	Clemente MI, Alvarez S, Serramía MJ, Martínez-Bonet M, Muñoz-Fernández MÁ	PLoS One. 2014 Feb 25;9(2):e85230	2014	3,234	(1st Quartile).
Carbosilane dendrimers as gene delivery agents for the treatment of HIV infection.	Perisé-Barrios AJ, Jiménez JL, Domínguez-Soto A, Javier de la Mata F, Corbí AL, Gomez R, Muñoz-Fernandez MA	J Control Release. 2014 Jun 28;184:51-7	2014	7,705	(1st Decilee)
Determinants of Highly Active Antiretroviral Therapy Duration in HIV-1-Infected Children and Adolescents in Madrid, Spain, from 1996 to 2012	Palladino C, Briz V, Bellón JM, Climent FJ, de Ory SJ, Mellado MJ, Navarro ML, Ramos JT, Taveira N, de José MI, Muñoz-Fernández MÁ; CoRISpeS-Madrid Cohort Working Group	PLoS One. 2014 May 1;9(5):e96307	2014	3,234	(1st Quartile).
Enhanced activity of carbosilane dendrimers against HIV when combined with reverse transcriptase inhibitor drugs: searching for more potent microbicides.	Vacas-Córdoba E, Galán M, de la Mata FJ, Gómez R, Pion M, Muñoz-Fernández MÁ.	Int J Nanomedicine. 2014 Jul 29;9:3591-600. doi: 10.2147/IJN.S62673. eCollection 2014.	2014	4,383	(1st Quartile).
Using CD4 Percentage and Age to Optimize Pediatric Antiretroviral Therapy Initiation	Dwight E. Yin, Meredith G. Warshaw, William C. Miller, Hannah Castro, Susan A. Fiscus, Lynda M. Harper, Linda J. Harrison, Nigel J. Klein, Joanna Lewis, Ann J. Melvin, Gareth Tudor-Williams and Ross E. McKinney Jr. PENPACT-1 (PENTA 9/PACTG 390) Study Team.	Pediatrics. 2014 Oct;134(4):e1104-16	2014	5,297	(1st Decilee)
HIV Infection Deregulates the Balance Between regulatory T Cells and IL-2–Producing CD4 T Cells by Decreasing the expression of the IL-2 Receptor in Treg	Gema Méndez-Lagares, Didiana Jaramillo-Ruiz, Marjorie Pion, Manuel Leal, M. A. Muñoz-Fernández, MD, Yolanda M. Pacheco, and Rafael Correa-Rocha	J Acquir Immune Defic Syndr. 2014 Mar	2014	4,425	(1st Quartile).
Shorter Telomere Length Predicts Poorer Immunological Recovery in Virologically Suppressed HIV-1 Infected Patients Treated with Combined antiretroviral Therapy.	Blanco JR, Jarrin I, Martínez A, Siles E, Larrayoz IM, Cañuelo A, Gutierrez F, Gonzalez-García J, Vidal F, Moreno S, CORIS-BIOBANCO.	J Acquir Immune Defic Syndr. 2015 Jan 1;68(1):21-9.	2015	4,556	(1st Quartile).
Conservation of g protein epitopes in respiratory syncytial virus (group a) despite broad genetic diversity: is antibody selection involved in virus evolution?	Trento A, Ábrego L, Rodriguez-Fernandez R, González-Sánchez MI, González-Martínez F, Delfraro A, Pascale JM, Arbiza J, Melero JA.	J Virol. 2015 Aug;89(15):7776-85	2015	4,439	(1st Quartile)
Clinical and virological follow-up in perinatally HIV-1 infected children and adolescents in Madrid with triple-class antiretroviral drug resistant viruses	Rojas P, de Mulder M, Fernandez-Cooke E, Prieto L, Rojo P, Jimenez de Ory S, Navarro M, Ramos JT and Holguin A, on behalf of the Madrid Cohort of HIV-infected children and adolescents integrated in the Pediatric branch of the Spanish National AIDS Network (CoRISPe).	Clinical Microbiology and Infection 2015: 21(6):605.e1-9.	2015	4,580	(1st Decilee)
Extremely high mutation rate of HIV-1 in vivo	Cuevas, J.M.; Geller, R.; Garijo, R.; López-Aldeguer, J.; Sanjuán, R.. PLoS Biol. 2015 Sep 16;13(9):e1002251.	Plos Biology. 13(9):e1002251.	2015	9,343	(1st Decilee)
Establishment and replenishment of the viral reservoir in perinatally HIV-1-infected children initiating very early antiretroviral therapy	Martínez-Bonet M, Puertas MC, Fortuny C, Ouchi D, Mellado J, Rojo P, Noguera-Julian A, Muñoz-Fernández MA, Martinez-Picado J	Clin Infect Dis. 2015 Oct 1;61(7):1169-78	2015	8,886	(1st Decilee)

Cardiac function in vertically HIV-infected children and adolescents in the era of highly active antiretroviral therapy	Sainz T, Álvarez-Fuente M, Fernández-Jiménez R, González-Tomé MI, de José MI, Ramos JT, Navarro ML, Martínez J, García-Hortelano M, Medrano C, Muñoz-Fernández MÁ, Mellado MJ; Madrid Cohort of HIV-Infected Children and Adolescents Integrated in the Pediatric Branch of the Spanish National AIDS Network (CoRISpeS)	Pediatr Infect Dis J. 2015 May;34(5):e125-31.	2015	3,135	(1st Decilee)
Primary resistance to integrase strand-transfer inhibitors in Europe.	Casadellà M, van Ham PM, Noguera-Julian M, van Kessel A, Pou C, Hofstra LM, Santos JR, Garcia F, Struck D, Alexiev I, Bakken Kran AM, Hoepelman AI, Kostrikis LG, Somogyi S, Liitsola K, Linka M, Nielsen C, Otelea D, Paraskevis D, Poljak M, Puchhammer-Stöckl E, Staneková D, Stanojevic M, Van Laethem K, Zidovec Lepej S, Clotet B, Boucher CA, Paredes R, Wensing AM; SPREAD programme..	J Antimicrob Chemother. 2015 Oct;70(10):2885-8	2015	4,919	(1st Decilee)
Use of carbosilane dendrimer to switch macrophage polarization for the acquisition of antitumor functions.	Perisé-Barrios AJ, Gómez R, Corbí AL, de la Mata J, Domínguez-Soto A, Muñoz-Fernandez MA	Nanoscale. 2015 Mar 7;7(9):3857-66	2015	7,394	(1st Decilee)
Safety and immunogenicity of a modified vaccinia Ankara-based HIV-1 vaccine (MVA-B) in HIV-1-infected patients alone or in combination with a drug to reactivate latent HIV-1	Mothe B, Climent N, Plana M, Rosàs M, Jiménez JL, Muñoz-Fernández MÁ, Puertas MC, Carrillo J, Gonzalez N, León A, Pich J, Arnaiz JA, Gatell JM, Clotet B, Blanco J, Alcamí J, Martinez-Picado J, Alvarez-Fernández C, Sánchez-Palomino S, Guardo AC, Peña J, Benito JM, Rallón N, Gómez CE, Perdiguero B, García-Arriaza J, Esteban M, López Bernaldo de Quirós JC, Brander C, García F; on behalf of the RISVAC-03 Study Group	J Antimicrob Chemother 2015; 70:1833-1842	2015	5,313	(1st Decilee)
Triple combination of carbosilane dendrimers, tenofovir and maraviroc as potential microbicide to prevent HIV-1 sexual transmission.	Sepúlveda-Crespo D, Sánchez-Rodríguez J, Serramía MJ, Gómez R, De La Mata FJ, Jiménez JL, Muñoz-Fernández MÁ.	Nanomedicine (Lond). 2015 Mar; 10(6):899-914.	2015	5,413	(1st Decilee)
Bryostatin activates HIV-1 latent expression in human astrocytes through a PKC and NF-κB-dependent mechanism.	Díaz L, Martínez-Bonet M, Sánchez J, Fernández-Pineda A, Jiménez JL, Muñoz E, Moreno S, Álvarez S, Muñoz-Fernández MÁ	Sci Rep. 2015 Jul 22;5:12442	2015	5,578	(1st Decilee)
Antiretroviral drugs do not interfere with bryostatin-mediated HIV-1 latency reversal	Martínez-Bonet M, Clemente MI, Álvarez S, Díaz L, García-Alonso D, Muñoz E, Moreno S, Muñoz-Fernández MÁ	Antiviral Res. 2015 Nov;123:163-71	2015	3,938	(1st Quartile).
Anti-Human Immunodeficiency Virus Activity of Thiol-Ene Carbosilane Dendrimers and Their PotentialDevelopment as a Topical Microbicide	Sánchez-Rodríguez J, Díaz L, Galán M, Maly M, Gómez R, Javier de la Mata F, Jiménez JL, Muñoz-Fernández MA.	J Biomed Nanotechnol. 2015 Oct;11(10):1783-98.	2015	5,338	(1st Quartile).
HIV-1 increases TLR responses in human primary astrocytes	Serramía MJ, Muñoz-Fernández MÁ, Álvarez S.	Sci Rep. 2015 Dec 16;5:17887	2015	3,057	(1st Quartile).
Synergistic Activation of Latent HIV-1 Expression by Novel Histone Deacetylase Inhibitors and Bryostatin-1	Marta Martínez-Bonet, Maria Isabel Clemente, Maria Jesús Serramía, Eduardo Muñoz, Santiago Moreno & Maria Ángeles Muñoz-Fernández.	Sci Rep. 2015 Nov 13;5:16445	2015	5,228	(1st Quartile).
A Phase I Randomized Therapeutic MVA-B Vaccination Improves the Magnitude and Quality of the T Cell Immune Responses in HIV-1-Infected Subjects on HAART	Gómez CE, Perdiguero B, García-Arriaza J, Cepeda V, Sánchez-Sorzano CÓ, Mothe B, Jiménez JL, Muñoz-Fernández MÁ, Gatell JM, López Bernaldo de Quirós JC, Brander C, García F, Esteban M.	PLoS One. 2015 Nov 6;10(11):e0141456	2015	3,234	(1st Quartile).
Description and consequences of prescribing off-label antiretrovirals in the Madrid Cohort of HIV-infected children over a quarter of century (1988-2012)	Fernandez.Cook E, Rojas P, Holguin A, Gonzalez-Tome I, Jimenez de Ory S, Mellado MJ, Navarro ML, Rojo P, Ramon JT.	Antivir Ther. 2016;21(1):65-70	2016	3,140	(2nd Quartile)
"Oxidative Stress Predicts All-Cause Mortality in HIV-Infected Patients	Masiá M, Padilla S, Fernández M, Rodríguez C, Moreno A, Oteo JA, Antela A, Moreno S, Del Amo J, Gutiérrez F; CoRIS, Biobanco.	PLoS One. 2016 Apr 25;11(4):e0153456.	2016	3,540	(1st Quartile)
Relationship between plasma bilirubin level and oxidative stress markers in HIV-infected patients on atazanavir- vs. efavirenz-based antiretroviral therapy	Estrada V, Monge S, Gómez-Garre MD, Sobrino P, Masiá M, Berenguer J, Portilla J, Viladés C, Martínez E, Blanco JR; CoRIS and the HIV Biobank integrated in the Spanish AIDS Research Network	HIV Med. 2016 Oct;17(9):653-61. IF: 3,341	2016	3,341	(2nd Quartile)
"Efficacy of HIV antiviral polyanionic carbosilane dendrimer G2-S16 in the presence of semen"	Ceña-Diez R, García-Broncano P, de la Mata FJ, Gómez R, Muñoz-Fernández MÁ.	Int J Nanomedicine. 2016 May 30;11:2443-50	2016	4,320	(1st Quartile)
Detection of Broadly Neutralizing Activity Within the First Months of HIV-1 Infection.	Sanchez-Merino V, Fabra-Garcia A, Gonzalez N, Nicolas D, Merino-Mansilla A, Manzardo C, Ambrosioni J, Schultz A, Meyerhans A, Mascola JR, Gatell JM, Alcami J, Miro JM, Yuste E.	J Virol. 2016 May 12;90(11):5231-45	2016	4,439	(1st Quartile).
Disease disclosure, treatment adherence, and behavioural profile in a cohort of vertically acquired HIV-infected adolescents. NeuroCoRISpeS study.	Medin G, García-Navarro C, Navarro Gomez M, Ramos Amador JT, Mellado MJ, Jimenez S, Muñoz-Fernández MA, Rojo Conejo P, Saavedra J, García Hortelano M, Guillén S, González-Tomé MI.	AIDS Care. 2016;28(1):124-30	2016	1,220	>2nd Quartile

Nanotechnology as a New Therapeutic Approach to Prevent the HIV-Infection of Treg Cells	Jaramillo-Ruiz D, De La Mata FJ, Gómez R, Correa-Rocha R, Muñoz-Fernández MÁ.	PLoS One. 2016 Jan 19;11(1):e0145760.	2016	2,806	(1st Quartile).
Antiviral mechanism of polyanionic carbosilane dendrimers against HIV-1	Enrique Vacas-Córdoba,Marek Maly,Francisco J De la Mata,Rafael Gómez,Marjorie Pion,Mª Ángeles Muñoz-Fernández	Int J Nanomedicine. 2016 Apr 5;11:1281-94	2016	4,300	(1st Quartile)
Balance between activation and regulation of HIV-specific CD8+ T-cell response after modified vaccinia Ankara B therapeutic vaccination	Rallón N, Mothe B, Lopez Bernaldo de Quiros JC, Plana M, Ligos JM, Montoya M, Muñoz-Fernández MA, Esteban M, Garcia F, Brander C, Benito JM; RISVAC03 Study Group	AIDS 2016; 30(4):553-562.	2016	5,019	(1st Quartile)
Validation of the HIV Tropism Test TROCAI Using the Virological Response to a Short-Term Maraviroc Monotherapy Exposure	Gonzalez-Serna A, Genebat M, De Luna-Romero M, Tarancon-Diez L, Dominguez-Molina B, Pacheco YM, Muñoz-Fernández MA, Leal M, Ruiz-Mateos E.	Antimicrob Agents Chemother. 2016 Sep 23;60(10):6398-401.	2016	4,302	(1st Quartile)
Dendronized PLGA nanoparticles with anionic carbosilane dendrons as antiviral agents against HIV infection	Galan M; Fornaguera C; Ortega P; Caldero G; Lorente R; Jimenez JL; de la Mata J; Munoz-Fernandez MA; Solans C; Gomez R.	RSC ADVANCES. 2016; 6(77) 73817-73826	2016	3,108	(2nd Quartile)
Rate and predictors of progression in elite and viremic HIV-1 controllers	Leon A, Perez I, Ruiz-Mateos E, Benito JM, Leal M, Lopez-Galindez C, Rallon N, Alcami J, Lopez-Aldeguer J, Viciana P, Rodriguez C, Grau E, Iribarren J, Gatell JM, Garcia F; EC and Immune Pathogenesis Working group of the Spanish AIDS Research Network.	AIDS 2016;30(8):1209-20. PMID: 26854807.	2016	4,407	(1st Quartile)
Improved Efficiency of Ibuprofen by Cationic Carbosilane Dendritic Conjugates	Perisé-Barrios AJ, Fuentes-Paniagua E, Sánchez-Nieves J, Serramía MJ, Alonso E, Reguera RM, Gómez R, de la Mata FJ, Muñoz-Fernández MÁ.	Mol Pharm. 2016 Oct 3;13(10):3427-3438	2016	4,440	(1st Quartile)
"Relationship between CCR5 (WT/Δ32) heterozygosity and HIV-1 reservoir size in adolescents and young adults with perinatally acquired HIV-1 infection"	Martínez-Bonet M, González-Serna A, Clemente MI, Morón-López S, Díaz L, Navarro M, Puertas MC, Leal M, Ruiz-Mateos E, Martinez-Picado J, Muñoz-Fernández MA.	Clin Microbiol Infect. 2017 May;23(5):318-324	2017	5,292	(1st Decilee)
"Higher levels of IL-6, CD4 turnover and Treg frequency are already present before cART in HIV-infected subjects with later low CD4 recovery". I Antivir Res (aceptado)	Isaac Rosado-Sánchez, Inmaculada Jarrín, María M. Pozo-Balado, Rebeca S. dE Pablo-Bernal, Inés Herrero-Fernández, Ana I. Alvarez-Ríos, Esther Rodríguez-Gallego, Miguel Genebat, Mar Vera, Juan Berenguer, María L. Martín, Enrique Bernal, Francesc Vodal, Julià Blanco, Manuel Leal and Yolanda M. Pacheco.	Antiviral Res. 2017 Jun;142:76-82.	2017	4,909	(1st Decilee)
"DNA sequences within glioma-derived extracellular vesicles can cross the intact blood-brain barrier and be detected in peripheral blood of patients"	García-Romero N, Carrión-Navarro J, Esteban-Rubio S, Lázaro-Ibáñez E, Peris-Celda M, Alonso MM, Guzmán-De-Villoria J, Fernández-Carballal C, de Mendivil AO, García-Duque S, Escobedo-Lucea C, Prat-Acín R, Belda-Iniesta C, Ayuso-Sacido A	Oncotarget. 2017 Jan 3;8(1):1416-1428.	2017	3,450	(1st Quartile)
"Contribution of Oxidative Stress to Non-AIDS Events in HIV-Infected Patients"	Masiá M, Padilla S, Fernández M, Barber X, Moreno S, Iribarren JA, Portilla J, Peña A, Vidal F, Gutiérrez F; CoRIS.	J Acquir Immune Defic Syndr. 2017 Jun 1;75(2):e36-e44	2017	3,806	(1st Quartile)
"Impact of lopinavir-ritonavir exposure in HIV-1 infected children and adolescents in Madrid, Spain during 2000-2014"	Rojas Sánchez P, Prieto L, Jiménez De Ory S, Fernández Cooke E, Navarro ML, Ramos JT, Holguín Á; Madrid Cohort of HIV-1 Infected Children and Adolescents Integrated in the Paediatric Branch of the Spanish National AIDS Network (CoRISPe)	PLoS One. 2017 Mar 28;12(3):e0173168.	2017	3,540	(1st Quartile)
"Thymic Function Failure Is Associated With Human Immunodeficiency Virus Disease Progression"	Ferrando-Martinez S, De Pablo-Bernal RS, De Luna-Romero M, De Ory SJ, Genebat M, Pacheco YM, Parras FJ, Montero M, Blanco JR, Gutierrez F, Santos J, Vidal F, Koup RA, Muñoz-Fernández MÁ, Leal M, Ruiz-Mateos E	Clin Infect Dis. 2017 May 1;64(9):1191-1197.	2017	8,736	(1st Decilee)
"HLA-B*57 and IFNL4-related polymorphisms are associated with protection against HIV-1 disease progression in controllers"	Dominguez-Molina B, Tarancon-Diez L, Hua S, Abad-Molina C, Rodriguez-Gallego E, Machmach K, Vidal F, Tural C, Moreno S, Goñi JM, Ramírez de Arellano E, Del Val M, Gonzalez-Escribano MF, Del Romero J, Rodriguez C, Capa L, Viciana P, Alcamí J, Yu XG, Walker BD, Leal M, Lichterfeld M, Ruiz-Mateos E; CRIS integrated in the Spanish AIDS Research Network.	Clin Infect Dis. 2017 Mar 1;64(5):621-628.	2017	8,736	(1st Decilee)
"Efficacy of carbosilane dendrimers with an antiretroviral combination against HIV-1 in the presence of semen-derived enhancer of viral infection"	García-Broncano P, Ceña-Diez R, de la Mata FJ, Gómez R, Resino S, Muñoz-Fernández MÁ.	Eur J Pharmacol. 2017 Sep 15;811:155-163. doi: 10.1016/j.ejphar.2017.05.060	2017	2,896	(2nd Quartile)
"IL7RA polymorphisms are not associated with AIDS progression"	Medrano LM, Jiménez JL, Jiménez-Sousa MA, Fernández-Rodíguez A, Gutiérrez-Rivas M, Bellón JM, Blanco JR, Inciarte A, Muñoz-Fernández MÁ, Resino S.	Eur J Clin Invest. 2017 Oct;47(10):719-727. doi: 10.1111/eci.12797. Epub 2017 Sep 2	2017	2,740	(1st Quartile)
"Impact of clinical parameters in the intra-host evolution of HIV-1 subtype B in pediatric patients".	Rojas Sánchez P, Cobos A, Navaro ML, Ramos JT, Pagán P and Holguín A.	Genome Biology and Evolution 2017 Oct 1;9(10):2715-2726.	2017	4,090	(1st Quartile)

"The Complexity of Antibody Responses Elicited against the Respiratory Syncytial Virus Glycoproteins in Hospitalized Children Younger than 2 Years".	Trento A, Rodríguez-Fernández R, González-Sánchez MI, González-Martínez F, Mas V, Vázquez M, Palomo C, Melero JA	Front Microbiol. 2017 Nov 22;8:2301. doi: 10.3389/fmicb.2017.02301. PMID: 29213258.	2017	4,076	(1st Quartile)
Factors Leading to the Loss of Natural Elite Control of HIV-1 Infection	Pernas M, Tarancón-Diez L, Rodríguez-Gallego E, Gómez J, Prado JG, Casado C, Dominguez-Molina B, Olivares I, Coiras M, León A, Rodriguez C, Benito JM, Rallón N, Plana M, Martinez-Madrid O, Dapena M, Iribarren JA, Del Romero J, García F, Alcamí J, Muñoz-Fernández MÁ, Vidal F, Leal M, Lopez-Galindez C, Ruiz-Mateos E. ECRIS integrated in the Spanish AIDS Research Network.	J Virol. 2017 Dec 6. pii: JVI.01805-17. doi: 10.1128/JVI.01805-17. [Epub ahead of print]	2017	4,663	(1st Quartile)
Lack of decline in hepatitis C virus incidence among HIV-positive men who have sex with men during 1990-2014.	van Santen DK, van der Helm JJ, Del Amo J, Meyer L, D'Arminio Monforte A, Price M, Béguelin CA, Zangerle R, Sannes M, Porter K, Geskus RB, Prins M; CASCADE Collaboration in EuroCoord	J Hepatol. 2017 Aug;67(2):255-262.	2017	12,486	(1st Decilee)
CD4 T cell decline following HIV seroconversion in individuals with and without CXCR4-tropic virus	Ghosn J, Bayan T, Meixenberger K, Tran L, Frange P, d'Arminio Monforte A, Zangerle R, de Mendoza C, Krastinova E, Porter K, Meyer L, Chaix ML; CASCADE Collaboration in EuroCoord.	J Antimicrob Chemother. 2017 Oct 1;72(10):2862-2868	2017	5,071	(1st Decilee)
New anionic carbosilane dendrons functionalized with a DO3A ligand at the focal point for the prevention of HIV-1 infection.	Moreno S, Sepúlveda-Crespo D, de la Mata FJ, Gómez R, Muñoz-Fernández MÁ.	Antiviral Res. 2017 Aug 18;146:54-64. doi: 10.1016/j.antiviral.2017.08.009.	2017	4,271	(1st Quartile)
New diagnoses of human immunodeficiency virus infection in the Spanish pediatric HIV Cohort (CoRISpe) from 2004 to 2013.	Jiménez de Ory S, González-Tomé MI, Fortuny C, Mellado MJ, Soler-Palacin P, Bustillo M, Ramos JT, Muñoz-Fernández MA, Navarro ML; Working groups of CoRISpe.	Medicine (Baltimore). 2017 Sep;96(39):e7858.	2017	1,803	2nd Quartile
Carbosilane dendrons with fatty acids at the core as a new potential microbicide against HSV-2/HIV-1 co-infection.	Guerrero-Beltrán C, Ceña-Diez R, Sepúlveda-Crespo D, De la Mata J, Gómez R, Leal M, Muñoz-Fernández MA, Jiménez JL.	Nanoscale. 2017 Nov 1. 2017 Nov 16;9(44):17263-17273.	2017	7,367	(1st Quartile)
Increased CD127+ and decreased CD57+ T cell expression levels in HIV-infected patients on NRTI-sparing regimens.	Gonzalez-Serna A, Ferrando-Martinez S, Tarancon-Diez L, De Pablo-Bernal RS, Dominguez-Molina B, Jiménez JL, Muñoz-Fernández MÁ, Leal M, Ruiz-Mateos E.	J Transl Med. 2017 Dec 20;15(1):259.	2017	3,786	(1st Quartile)
Study of non-covalent interactions on dendriplex formation: Influence of hydrophobic, electrostatic and hydrogen bonds interactions	Sánchez-Milla M, Pastor I, Maly M, Serramía MJ, Gómez R, Sánchez-Nieves J, Ritort F, Muñoz-Fernández MÁ, de la Mata FJ.	Colloids Surf B Biointerfaces. 2018 Feb 1;162:380-388.	2017	3,887	(1st Quartile)
"Expression profiling of chromatin-modifying enzymes and global DNA methylation in CD4+ T cells from patients with chronic HIV infection at different HIV control and progression states"	Bogoi RN, de Pablo A, Valencia E, Martín-Carbonero L, Moreno V, Vilchez-Rueda HH, Asensi V, Rodriguez R, Toledano V, Rodés B.	Clin Epigenetics. 2018 Feb 13;10:20. doi: 10.1186/s13148-018-0448-5.	2018	2,900	(2nd Quartile)
"Early and Highly Suppressive Antiretroviral Therapy Are Main Factors Associated With Low Viral Reservoir in European Perinatally HIV-Infected Children".	Tagarro A, Chan M, Zangari P, Ferns B, Foster C, De Rossi A, Nastouli E, Muñoz-Fernández MA, Gibb D, Rossi P, Giaquinto C, Babiker A, Fortuny C, Freguja R, Cotugno N, Judd A, Noguera-Julian A, Navarro ML, Mellado MJ, Klein N, Palma P, Rojo P.	J Acquir Immune Defic Syndr. 2018 Oct 1;79(2):269-276.	2018	4,116	(1st Quartile)
"Elevated liver stiffness is linked to increased biomarkers of inflammation and immune activation in HIV/hepatitis C virus-coinfected patient"	Medrano LM, Garcia-Broncano P, Berenguer J, González-García J, Jiménez-Sousa MÁ, Guardiola JM, Crespo M, Quereda C, Sanz J, Canorea I, Carrero A, Hontañón V, Muñoz-Fernández MÁ, Resino S.	AIDS. 2018 Jun 1;32(9):1095-1105.	2018	5,019	(1st Quartile)
"Class-modeling analysis reveals T-cell homeostasis disturbances involved in loss of immune control in elite controllers. "	José M Benito, María C Ortiz, Agathe León, Luis A Sarabia, José M Ligos, María Montoya, Marcial García, Ezequiel Ruiz-Mateos, Rosario Palacios, Alfonso Cabello, Clara Restrepo, Carmen Rodriguez, Jorge del Romero, Manuel Leal, María A Muñoz-Fernandez, José Alcamí, Felipe García, Miguel Górgolas, Norma Rallón, and On behalf of ECRIS integrated in the Spanish AIDS Research Network.	BMC Med. 2018 Feb 28;16(1):30. doi: 10.1186/s12916-018-1026-6.	2018	8,100	(1st Decilee)
"High plasma levels of stnf-r1 and ccl11 are related to CD4+ T-cells fall in HIV elite controllers with a sustained virologic control".	Mónica Gutiérrez-Rivas, María Ángeles Jiménez-Sousa, Norma Rallón, José Luis Jíménez, Clara Restrepo, Agathe León, Marta Montero-Alonso, Juan González-García, María Ángeles Muñoz-Fernández, José Miguel Benito, Salvador Resino.	Front Immunol. 2018 Jun 18;9:1399. doi: 10.3389/fimmu.2018.01399. eCollection 2018.	2018	6,430	(1st Quartile)
"Role of APOBEC3H in the viral control of HIV Elite Controller Patients".	José M Benito, Julia Hillung, Clara Restrepo, José M Cuevas, Agathe León, Ezequiel Ruiz-Mateos, Rosario Palacios-Muñoz, Miguel Górgolas, Rafel Sanjuán, Norma Rallón; On behalf of ECRIS integrated in the Spanish AIDS Research Network.	Int J Med Sci. 2018 Jan 1;15(2):95-100. doi: 10.7150/ijms.22317. eCollection 2018.	2018	2,400	(1st Quartile)

"Lower expression of plasma-derived exosome miR-21 levels in elite controllers with decreasing CD4 T cell count".	María J Ruiz-de-León, María A Jiménez-Sousa, Santiago Moreno, Marcial García, Mónica Gutierrez-Rivas, Agathe León, Marta Montero-Alonso, Juan González-García, Salvador Resino, Norma Rallón, José M Benito, Alejandro Vallejo, On behalf of the ECRIS Network integrated in the Spanish AIDS research network.	J Microbiol Immunol Infect. 2018 Aug 24. pii: S1684-1182(18)30308-6. doi: 10.1016/j.jmii.2018.07.007. [Epub ahead of print]	2018	2,094	(1st Quartile)
Characterization of broadly neutralizing antibody responses to HIV-1 in a cohort of long term non-progressors.	González N, McKee K, Lynch RM, Georgiev IS, Jimenez L, Grau E, Yuste E, Kwong PD, Mascola JR y Alcamí J.	PLoS One. 2018 Mar 20;13(3):e0193773. doi: 10.1371/journal.pone.0193773.	2018	3,540	(1st Quartile)
Dysregulation of the immune system in HIV/HCV-coinfected patients according to liver stiffness status	García-Broncano P, Medrano LM, Berenguer J, Gonzalez-García J, Jiménez-Sousa MA, Carrero A, Hontañón V, Guardiola JM, Crespo M, Quereda C, Sanz J, García-Gomez A, Jimenez JL, Resino S	Cells 2018, 7(11):196.	2018	4,830	(2nd Quartile)
Mitochondrial haplogroup H is related to CD4+ T cell recovery in HIV infected patients starting combination antiretroviral therapy	Luz M Medrano, Mónica Gutiérrez-Rivas, Julià Blanco, Marcial García, María A Jiménez-Sousa, Yolanda M Pacheco, Marta Montero, José Antonio Iribarren, Enrique Bernal, Onofre Juan Martínez, José M Benito, Norma Rallón, S Resino and CoRIS and the HIV Biobank integrated in the Spanish AIDS Research Network Project RIS/EPICLIN 10_2015	Transl Med 2018; 16:343.	2018	4,200	(1st Quartile)
HIV-Infected Subjects With Poor CD4 T-Cell Recovery Despite Effective Therapy Express High Levels of OX40 and α4β7 on CD4 T-Cells Prior Therapy Initiation.	Rosado-Sánchez I, Herrero-Fernández I, Genebat M, Del Romero J, Riera M, Podzamczer D, Olalla J, Vidal F, Muñoz-Fernández MA, Leal M, Pacheco YM	Front Immunol 2018; 9: 1673.	2018	4,716	(1st Quartile)
Increased frequencies of Th17 cells and IL17a-producing regulatory T-cells preceding the immunodiscordant response to antiretroviral treatment.	Rosado-Sánchez I, Herrero-Fernández I, Tarancon-Diez L, Moreno S, Iribarren JA, Dalmau D, Vera-Méndez F, Leal M, Pacheco YM.	J Infect 2018; 76: 86-92	2018	5,099	(1st Quartile)
Trends in drug resistance prevalence, HIV-1 variants and clinical status in HIV-1- infected paediatric population in Madrid: 1993-2015 Analysis	Rojas Sánchez P, Dominguez S, Jiménez de Ory S, Prieto L, Rojo P, Mellado MJ, Navarro M, Delgado R, Ramos JT, and Holguín A., on behalf of the Madrid Cohort of HIV-1 Infected Children and Adolescents Integrated in the Paediatric Branch of the Spanish National AIDS Network (CoRISPe	The Pediatrics Infectious Disease Journal. 2018 Mar;37(3):e48-e57. doi: 10.1097/INF.0000000000001760	2018	2,723	(1st Quartile).
Effect of HIV/HCV Co-Infection on the Protease Evolution of HIV-1B: A Pilot Study in a Pediatric Population	Domínguez-Rodríguez S, Rojas P, Fernández McPhee C, Pagán I, Navarro ML, Ramos JT, Holguín Á	Sci Rep. 2018 Feb 5;8(1):2347. doi: 10.1038/s41598-018-19312-2	2018		(1st Quartile).
Sulfonate-ended carbosilane dendrimers with a flexible scaffold cause inactivation of HIV-1 virions and gp120 shedding	Sepúlveda-Crespo D, de la Mata FJ, Gómez R, Muñoz-Fernández MA	Nanoscale. 2018 May 17;10(19):8998-9011.	2018	7,367	(1st Quartile)
High Plasma Levels of sTNF-R1 and CCL11 Are Related to CD4+ T-Cells Fall in Human Immunodeficiency Virus Elite Controllers With a Sustained Virologic Control.	Gutiérrez-Rivas M, Jiménez-Sousa MÁ, Rallón N, Jiménez JL, Restrepo C, León A, Montero-Alonso M, González-García J, Muñoz-Fernández MÁ, Benito JM, Resino S; ECRIS Integrated in the Spanish AIDS Research Network.	Front Immunol.2018 Jun 18;9:1399. doi: 10.3389/fimmu.2018.01399. eCollection 2018.	2018	5,511	(1st Quartile)
HIV-Infected Subjects With Poor CD4 T-Cell Recovery Despite Effective Therapy Express High Levels of OX40 and α4β7 on CD4 T-Cells Prior Therapy Initiation.	Rosado-Sánchez I, Herrero-Fernández I, Genebat M, Del Romero J, Riera M, Podzamczer D, Olalla J, Vidal F, Muñoz-Fernández MA, Leal M, Pacheco YM.	Front Immunol.2018 Jul 18;9:1673. eCollection 2018.	2018	5,511	(1st Quartile)
Immediate vs. Deferred Switching from a Boosted Protease Inhibitor (PI/r) Based Regimen to a Dolutegravir (DTG) Based Regimen in Virologically Suppressed Patients with High Cardiovascular Risk or Age ≥50 years: Final 96 Weeks Results of NEAT 022 study	Gatell JM, Assoumou L, Moyle G, Waters L, Johnson M, Domingo P, Fox J, Martinez E, Stellbrink HJ, Guaraldi G, Masia M, Gompels M, De Wit S, Florence E, Esser S, Raffi F, Stephan C, Rockstroh J, Giacomelli A, Vera J, Bernardino JI, Winston A, Saumoy M, Gras J, Katlama C, Pozniak AL; NEAT022 Study Group.	Clin Infect Dis. 2018 Jun 14. doi: 10.1093/cid/ciy505. [Epub ahead of print]	2019	9,110	(1st Decilee)
Proteomic Profile Associated with Loss of Spontaneous HIV-1 Elite Control.	Rodríguez-Gallego E, Tarancón-Diez L, García F, Del Romero J, Miguel Benito J, Alba V, Herrero P, Rull A, Dominguez-Molina B, Martinez-Madrid O, Martin-Pena L, Pulido F, León A, Rodríguez C, Rallón N, Peraire J, Viladés C, Leal M, Vidal F, Ruiz-Mateos E; ECRIS integrated in the Spanish AIDS Research Network.	J Infect Dis. 2018 Oct 12. doi: 10.1093/infdis/jiy599. [Epub ahead of print]	2019	5,189	(1st Quartile)
VDR rs2228570 Polymorphism Is Related to Non-Progression to AIDS in Antiretroviral Therapy Naïve HIV-Infected Patients.	Jiménez-Sousa MA, Jiménez JL, Fernández-Rodríguez A, Brochado-Kith O, Bellón JM, Gutierrez F, Díez C, Bernal-Morell E, Viciana P, Muñoz-Fernández MA, Resino S.	J Clin Med. 2019 Mar 5;8(3)	2019	5,583	(1st Decilee)

Massive release of platelet-derived extracellular vesicles and mitochondrial dysfunction among elite controllers, viremic controllers and ART-virological suppressed HIV-infected patients: New biomarkers of HIV pathogenesis?	Poveda E, Tabernilla A, Salgado A, et al.	Clinical Infectious Diseases 2019, (submitted July 2019).	2019	9,055	(1st Decilee)
Genetic variation in CCR2 and CXCL12 genes impacts on CD4 restoration in patients initiating cART with advanced immunesupression.	Restrepo C, Gutierrez-Rivas M, Pacheco YM, García M, Blanco J, Medrano LM, Navarrete-Muñoz MA, Gutiérrez F, Miralles P, Dalmau D, Gómez JL, Górgolas M, Cabello A, Resino S, Benito JM, Rallón N; CoRIS and the HIV Biobank integrated in the Spanish AIDS Research Network Project RIS/EPICLIN 10_2015.	PLoS One. 2019 Mar 28;14(3):e0214421.PMID: 30921390.	2019	2,770	(1st Quartile)
Glutaminolysis and lipoproteins are key factors in late immune recovery in successfully treated HIV-infected patients	Rosado-Sánchez I, Rodríguez-Gallego E, Peraire J, Viladés C, Herrero P, Fanjul F, Gutiérrez F, Bernal E, Pelazas R, Leal M, Veloso S, López-Dupla M, Blanco J, Vidal F, Pacheco YM, Rull A.	Clin Sci 2019; 133: 997-1010.	2019	5,237	(1st Quartile)
IL7RA rs6897932 Polymorphism is Associated with Better CD4+ T-Cell Recovery in HIV Infected Patients Starting Combination Antiretroviral Therapy	Resino S, Navarrete-Muñoz MA, Blanco J, Pacheco YM, Castro I, Berenguer J, Santos J, Vera-Méndez FJ, Górgolas M, Jiménez-Sousa MAÁ, Benito JM, Rallón N; CoRIS and the HIV Biobank integrated in the Spanish AIDS Research Network Project RIS/EPICLIN 10_2015.	Biomolecules 2019; 9: pii: E233.	2019	4,694	(1st Quartile)
Lower expression of plasma-derived exosome miR-21 levels in HIV-1 elite controllers with decreasing CD4 T cell count.	Ruiz-de-León MJ, Jiménez-Sousa MA, Moreno S, García M, Gutiérrez-Rivas M, León A, Montero-Alonso M, González-García J, Resino S, Rallón N, Benito JM, Vallejo.	J Microbiol Immunol Infect. 2019; S1684-1182(18)30308-6.	2019	2,460	(3rd Quartile)
VDR rs2228570 polymorphism is related to non-progression to AIDS in antiretroviral therapy naïve HIV-infected patients.	Jiménez-Sousa MA, Jiménez JL, Fernández-Rodríguez A, Brochado-Kith O, Bellón JM, Gutiérrez F, Díez C, Bernal-Morell E, Viciana P, Muñoz-Fernández MA, Resino S (*).	J Clin Med. 2019. 8(3): e311. PMID: 30841566.	2019	5,690	(1st Decilee)
Prevalence of hepatitis E infection in HIV/HCV-coinfected patients in Spain (2012-2014).	Vázquez-Morón S, Berenguer J, González-García J, Jiménez-Sousa MA, Guardiola JM, Crespo M, Quereda C, Sanz J, Carrero A, Hontañón V, Avellón A, Resino S (*).	Sci Rep. 2019; 9(1): 1143.	2019	4,010	(1st Quartile)
Genetic variants upstream of TNFAIP3 in the 6q23 region are associated with liver disease severity in HIV/HCV-coinfected patients: a cross-sectional study.	Jiménez-Sousa MA, Berenguer J, Fernández-Rodríguez A; Medrano LM, Aldámiz-Echevarria T, Pérez-Latorre L, Díez C, Martín-Vicente M, Gutiérrez-Rivas M, Martínez I, Resino S (*).	Infect Genet Evol 2019; 67:112-120.	2019	2,610	(3rd Quartile)
Surveillance of transmitted drug resistance to integrase inhibitors in Spain: implications for clinical practice.	Alvarez M, Casas P, de Salazar A, Chueca N, Guerrero-Beltran C, Rodríguez C, Imaz A, Espinosa N, García-Bujalance S, Pérez-Elías MJ, García-Alvarez M, Iribarren JA, Santos J, Dalmau D, Aguilera A, Vinuesa D, Gutiérrez F, Piérola B, Molina JM, Peraire J, Portilla I, Gómez-Sirvent JL, Olalla J, Galera C, Blanco JR, Riera M, García-Fraile L, Navarro G, Curran A, Poveda E, García F; CoRIS .	J Antimicrob Chemother.	2019	5,113	(1st Decilee)
G2-S16 dendrimer microbicide does not interfere with the vaginal immune system.	Martín-Moreno A, Sepúlveda-Crespo D, Serramía-Lobera MJ, Perisé-Barrios AJ, Muñoz-Fernández MA.	J Nanobiotechnology. 2019 May 15;17(1):65	2019	5,924	(1st Quartile)
G1-S4 or G2-S16 carbosilane dendrimer in combination with Platycodin D as a promising vaginal microbicide candidate with contraceptive activity.	Ceña-Diez R, Martin-Moreno A, de la Mata FJ, Gómez-Ramirez R, Muñoz E, Ardoy M, Muñoz-Fernández MÁ.	Int J Nanomedicine. 2019 Apr 2;14:2371-2381	2019	4,370	(1st Quartile)
Poly(N-vinylcaprolactam) Nanogels with Antiviral Behavior against HIV-1 Infection.	Macchione MA, Guerrero-Beltrán C, Rosso AP, Euti EM, Martinelli M, Strumia MC, Muñoz-Fernández MÁ.	Sci Rep. 2019 Apr 5;9(1):5732.	2019	4,122	(1st Decilee)
VDR rs2228570 Polymorphism Is Related to Non-Progression to AIDS in Antiretroviral Therapy Naïve HIV-Infected Patients	Jiménez-Sousa MA, Jiménez JL, Fernández-Rodríguez A, Brochado-Kith O, Bellón JM, Gutierrez F, Díez C, Bernal-Morell E, Viciana P, Muñoz-Fernández MA, Resino S.	J Clin Med. 2019 Mar 5;8(3).	2019	5,583	(1st Decilee)
Transcriptome Sequencing Of Peripheral Blood Mononuclear Cells From Elite Controller-Long Term Non Progressors	Francisco Díez Fuertes, Humberto Erick de la Torre Tarazona,, Esther Calonge, Maria Pernas, Javier García-Pérez, Laura Capa, Anavaj Sakuntabhai, José Alcamí	Sci Rep. 2019 Oct 3;9(1):14265. doi: 10.1038/s41598-019-50642-x.	2019	4,010	(1st Quartile)
CD4 recovery is related to genetic variation in genes of immune response	Marcial García, Luz M Medrano, Julià Blanco, Clara Restrepo, Yolanda M Pacheco, Mónica GutierrezRivas, María A Navarrete-Muñoz, Juan Carlos López-Bernaldo, Félix Gutiérrez, Joaquín Portilla, Vicente Estrada, Miguel Górgolas, Alfonso Cabello, Salvador Resino, José Miguel Benito, Norma Rallón; on behalf of CoRIS and the Spanish HIV Biobank integrated in the Spanish AIDSResearch Network Project RIS/EPICLIN 10_2015	Antiviral Res. Under review	2019	4,130	(1st Quartile)
